# Spatio-temporal independent component classification for localization of seizure onset zone

**DOI:** 10.3389/fneur.2025.1515484

**Published:** 2025-06-06

**Authors:** Seyyed Mostafa Sadjadi, Elias Ebrahimzadeh, Alireza Fallahi, Jafar Mehvari Habibabadi, Mohammad-Reza Nazem-Zadeh, Hamid Soltanian-Zadeh

**Affiliations:** ^1^Control and Intelligent Processing Center of Excellence (CIPCE), School of Electrical and Computer Engineering, College of Engineering, University of Tehran, Tehran, Iran; ^2^Neuroimage Signal and Image Analysis Group, School of Cognitive Sciences, Institute for Research in Fundamental Sciences (IPM), Tehran, Iran; ^3^Department of Biomedical Engineering, Hamedan University of Technology, Hamedan, Iran; ^4^Isfahan Neuroscience Research Center, Isfahan University of Medical Sciences, Isfahan, Iran; ^5^Department of Medical Physics and Biomedical Engineering, Tehran University of Medical Sciences, Tehran, Iran; ^6^Department of Neuroscience, Monash University, Melbourne, VIC, Australia

**Keywords:** epilepsy, epileptogenic zone, source localization, fMRI, independent component analysis (ICA), functional connectivity (FC), local network features

## Abstract

Localization of the epileptic seizure onset zone (SOZ) as a step in presurgical planning leads to higher efficiency in surgical and stimulation treatments. However, the clinical localization procedure is a difficult, long procedure with increasing challenges in patients with complex epileptic foci. The interictal methods have been proposed to assist in presurgical planning with simpler procedures for data acquisition and higher speeds. In this study, spatio-temporal component classification (STCC) is presented for the localization of epileptic foci using resting-state functional magnetic resonance imaging (rs-fMRI) data. This method is based on spatio-temporal independent component analysis (ST-ICA) on rs-fMRI data with a component-sorting procedure based on the dominant power frequency, biophysical constraints, spatial lateralization, local connectivity, temporal energy, and functional non-Gaussianity. STCC was evaluated in 13 patients with temporal lobe epilepsy (TLE) who underwent surgical resection and had seizure-free surgical outcomes after a 12-month follow-up. The results showed promising accuracy, highlighting valuable features that serve as SOZ functional biomarkers. Unlike most presented methods, which depend on simultaneous EEG information, the occurrence of epileptic spikes, and the depth of the epileptic foci, the presented method is entirely based on fMRI data making it independent of such information, simpler to use in terms of data acquisition and artifact removal, and considerably easier to implement.

## Highlights

- Novel fMRI-only method localizes epileptic foci for presurgical evaluation.
- Uses spatio-temporal ICA features to classify components from rs-fMRI data.
- Achieves high accuracy without EEG, seizure events, or depth limitations.
- Evaluated in 13 unilateral TLE patients with seizure-free post-surgical outcomes.
- Simplifies data acquisition, making the method accessible, fast, and non-invasive.

## 1 Introduction

Epilepsy is one of the most widespread neurological disorders (1) causing spontaneous seizures as a result of excessive, abnormal, or synchronous neuronal activities (2). The seizures can originate from one or several specific zones or generalize over the brain tissue. Using anti-seizure drugs, seizures can be controlled, but can also be drug-resistant in about 20–30% of cases (3). For refractory epilepsy, surgical resection is among the well-established approaches to control seizures (4). However, presurgical planning should include a localization step for epileptic foci. This allows the surgeon to orient the skull opening and proceed more efficiently with the surgical steps. Removal of the epileptogenic zone may lead to cognitive deficits, particularly when the resection areas are associated with working memory, attention, or executive functions. Targeted rehabilitation helps regain lost functions or compensating for them through neural plasticity (5–8). Cognitive therapies and neurostimulation techniques have shown promise in enhancing recovery and improving patients' quality of life post-surgery. They also allow better integration into daily activities and social environments (9–12).

In this work, we refer to an epileptic focus as the specific brain region where seizures originate (often identified as the seizure onset zone on EEG or imaging). An epileptic network denotes the broader set of interconnected regions that participate in seizure generation and propagation beyond the initial focus (13). In contrast, the epileptogenic zone (EZ) is the brain area indispensable for seizure generation—the region that must be completely removed or neutralized to achieve seizure freedom. The seizure onset zone (SOZ) is typically used as the best available estimator of the EZ in clinical practice (14). By clearly defining these terms, we avoid confusion. Our method aims to localize the SOZ (epileptic focus) non-invasively, which is assumed to closely approximate the epileptogenic zone in our cohort of temporal lobe epilepsy (TLE) patients.

Various methods using ictal, interictal, invasive, and non-invasive data recordings, have been developed to localize the generation area, spatial extent, and propagation pathways of epileptic activity. These approaches face a constant challenge to balance accuracy against the simplicity of data acquisition and implementation (15). The current gold standard for localizing epileptic foci relies on ictal (seizure) onset, which requires recording multiple typical seizures from the patient (16). However, seizure frequency is relatively low compared to interictal epileptiform discharges (IEDs). Invasive approaches such as intracranial electroencephalography (iEEG) offer high yet local spatial resolution. They are therefore used to localize the seizure focus and define the epileptogenic zone (EZ) during later steps of presurgical planning (17). Despite the time-consuming process of the clinical protocol, the success rate of the resection surgery varies depending on several factors. Post-surgical follow-ups can range from a few months to more than 5 years. Surgical outcomes are classified by the Engel criteria (18): Engel I for seizure freedom (about 50–70% of cases); Engel II with warning signs or minor seizures for < 3 days per year (about 10–30% of cases); Engel III for a >80% reduction in seizure frequency or worthwhile improvement in the seizure-related disability (about 10–30% of cases); and Engel IV for < 80% reduction in seizure frequency or no worthwhile improvement (< 10% of cases) (4).

Although localizing epileptic foci from seizure-free (interictal) periods remains challenging, especially in the absence of IEDs, non-invasive methods have gained attention in recent years, especially those based on EEG-correlated fMRI (EEG-fMRI) (19, 20). This combination benefits from the high temporal resolution of the EEG signal and the high spatial resolution of blood oxygen level-dependent (BOLD) fMRI at the same time. Khoo et al. (21) showed that an IED adjacent to a maximum BOLD response, which often corresponds to the seizure onset zone, is more likely to precede IEDs in remote locations during widespread intracranial discharges. Therefore, simultaneous EEG-fMRI is a unique non-invasive method to reveal the origin of IEDs. Notably, localizing epileptic foci with these approaches is a key step in presurgical planning, as it provides surgeons with an approximate region of interest for opening the skull. The final resection is then guided by precise intracranial electrode investigation during surgery.

The conventional approach for localizing epileptic foci using simultaneous EEG-fMRI is based on statistical parametric mapping (SPM). It simply assumes IEDs found in the EEG signal as zero-duration events and uses their timing to generate a regressor for the general linear model (GLM) analysis on the simultaneous fMRI. Activations and deactivations can be localized for a regressor in GLM referring to positive and negative BOLD responses, respectively. The concordance with IEDs, however, seems to be more associated with positive BOLD responses and less associated with the deactivations (22). The study by Zijlmans et al. (23) is an important work on the conventional method with 29 patients, who were rejected for surgery because of multifocality or unclear foci, and 46 IED sets to study. They found a considerable improvement in localizing epileptic foci during the presurgical assessment. Eight patients showed BOLD responses topographically related to their IEDs. In a commentary on this study using 49 IED sets from 29 patients, Jackson et al. (24) revealed 15 BOLD responses, providing new predictions for surgery. However, it is noticeable that IEDs may generally be revealed in the brain regions well beyond the presumed area in which they are generated (25), and are not a perfect option as a base for concordance evaluation.

De Tiège et al. (26) recruited six children with refractory focal epilepsy and analyzed their EEG-fMRI data using the conventional method. In four children, the results showed BOLD responses concordant with the assumed epileptic foci. In another study, Zhang et al. (27) investigated the results of pre-surgical conventional EEG-fMRI analysis and iEEG monitoring in a patient with seizure recurrence after epilepsy surgery. They suggested that EEG-fMRI is a useful tool for pre-surgical evaluation but requires caution. The intact seizure foci in the remaining brain may also cause a non-seizure-free outcome.

In the localization of epileptic foci, combining two neuroimaging modalities has generated more accurate results than a single modality (28, 29). Ebrahimzadeh et al. (8) showed that localizing based on EEG data alone even when using independent component analysis (ICA) leads to poor results. However, localization of epileptic foci based on fMRI data alone is still receiving attention. Several studies argue that the relationships between BOLD and local field potential (LFP) are not always linear and may change depending on various factors. It has been shown that functional connectivity (FC) measured by BOLD and EEG signals have relatively weak correlations (30). Not many studies have investigated the neural basis of such spontaneous fluctuations in fMRI signals (31, 32). These two modalities measure different phenomena related to epilepsy possibly occurring at different time scales. We might see specific electrophysiological features of epileptic networks in stereotactic EEG (SEEG), while rs-fMRI normally reflects the functionality of such networks (30).

The influence of EEG spikes on BOLD signals is not quite clear (30). Simultaneous EEG-fMRI recordings have revealed that spikes can lead to increased, decreased, or unchanged BOLD signals (33, 34). The hypothesis of neurovascular decoupling has also been questioned as explain this complexity (35). EEG-fMRI is generally limited by the detection of frequent spikes on scalp EEG and the underestimation of those not expressed properly on surface EEG may be due to sources in deep brain structures (12, 30, 36–38). This can cause a false BOLD signal baseline. IEDs may themselves be at least in part responsible for the discrepancy between EEG and BOLD coupling, but already found negative correlations between these two signals in regions spared by epileptiform abnormalities suggest that spikes are not solely responsible (30).

There are useful tools to analyze and study abnormal activities with specific sources within the brain volume. The idea of exploring fMRI data for candidate sources related to epilepsy was discussed by Zhang et al. (39) and more recently by Banerjee et al. (40) who analyzed components obtained from spatial ICA (sICA). Temporal clustering analysis (TCA) (41), temporal ICA (tICA) (42), and fractional power spectrum contribution (fPSC) (43) are also shown to be useful to find and confirm epileptic activity. Moreover, functional connectivity analysis is a promising way to look for the functionally integrated relationships within a cluster and between spatially separated brain regions. This can be helpful to define local network topological features, localize the significantly connected areas to the SOZ, and identify the propagation pathways of epileptic activity (44). In Table 1, pivotal studies that present interictal methods for epileptic foci localization using EEG, fMRI, and EEG-fMRI and include seizure-free surgical outcomes for evaluation are reported chronologically. The methods mentioned might have been used and evaluated in other studies after their presentation as well, in different groups of patients.

**Table 1:** Studies presenting epileptic foci localization methods using EEG, fMRI, and EEG-fMRI data and including seizure-free surgical outcome data for evaluation.

**Study**	**Year**	**Sample size**	**Method data**	**Method**	**Accuracy/results**
Brodbeck et al. (57)	2010	10	EEG	Eelctrical source imaging (ESI) using LAURA on the IEDs.	8/9 (88%) within the resection margins.
Thornton et al. (58)	2010	34	EEG-fMRI	Conventional EEG-fMRI method using the extracted timing of IEDs convolved with canonical hemodynamic response function (HRF) as the regressor for GLM on fMRI data.	10/34 (30%) had surgical resection and significant activation on EEG-fMRI, 7/10 were seizure-free following surgery, and 6/7 had concordant results with resection.
Grouiller et al. (59)	2011	23	EEG-fMRI	The EEG voltage map of the IED template was correlated with Intra-MRI EEG voltage maps to construct the epileptic activity for further GLM analysis.	10/18 (55%) fully concordant and 4/18 (22%) were moderately concordant to the postoperative areas with seizure freedom.
Hauf et al. (51)	2012	10	EEG-fMRI	Three different threshold criteria were applied to detect hemodynamic responses to the IEDs: peak activation (criterion 1), fixed threshold at *P < * 0.05 corrected for multiple comparison (criterion 2), and fixed numbers of activated voxels (4,000 ± 200) within the brain (criterion 3).	5/10 (50%) concordance with criterion 1, 6/10 (60%) concordance with criterion 2, 8/10 (80%) concordance with criterion 3.
Pouliot et al. (60)	2012	3	EEG-fMRI	Non-linear hemodynamic responses using the second-order expansion of the Volterra kernel with epileptic spikes as time-dependent inputs and BOLD, oxyhemoglobin (HbO), and deoxyhemoglobin (HbR) time series at a certain fMRI voxel as the outputs.	3/3 (100%) concordance of significant non-linearities with the epileptic foci and negative BOLD response regions.
An et al. (61)	2013	35	EEG-fMRI	Conventional method with combined t map of four HRFs peaking at three, five, seven, and 9 s.	10/35 (29%) fully lobe concordant, 9/35 (26%) partially lobe concordant, 5/35 (14%) partially lobe discordant, 11/35 (31%) fully lobe discordant.
van Houdt et al. (62)	2015	8	fMRI	sICA was applied to two fMRI data epochs with and without visible IEDs separately and the epileptic sIC was found using spatial correlation with the resection area and EEG-fMRI correlation patterns.	7/8 (88%) remarkable resemblance between the epileptic sICs in the two states suggesting that epilepsy-related sICs are not dependent on the presence of IEDs.
Hunyadi et al. (63)	2015	12	EEG-fMRI	Most correlated sICs with resection areas were obtained from EEG tICA and fMRI sICA, and correlation coefficients were calculated for all possible pairs of EEG-eICs convolved with HRF and fMRI-eICs.	3/12 (25%) epileptic sICs were matched between EEG and fMRI and overlapped to the epileptic zone.
Zhang et al. (39)	2015	9	fMRI	sICA was applied to fMRI data extracting 30 sICs and the epileptic sIC was found using biophysical constraints, temporal features, and lateralization index.	7/9 (78%) lobe concordant results.
Coan et al. (64)	2016	30	EEG-fMRI	The regressor proposed in Grouiller et al. (49) plus the conventional regressor were used together in GLM analysis.	81% sensitivity and 79% specificity of results to identify patients with good surgical outcome.
Centeno et al. (65)	2017	53	EEG and EEG-fMRI	EEG-fMRI global maxima (GM) along with ESI.	17/53 (32%) localized by ESI, 11/53 (21%) localized by GM, and 11/53 (21%) localized by both with the mean distance of 14.6 mm in their maxima.
Maziero et al. (66)	2018	18	EEG-fMRI	2dTCA presented for mapping the seizure onset zone.	13/18 (72%) concordant results not confined to the presence of IEDs.
Chaudhary et al. (67)	2021	8	iEEG-fMRI	38 different topographic IEDs were classified and extracted from iEEG and BOLD changes associated with individual IED classes were assessed over the whole brain using GLM.	27/38 (71%) IED classes resulted in concordant BOLD maps.

According to Table 1, localization methods using interictal data have been tested for several years. From a major investigation (19), only 23% of the presented methods in the literature have been evaluated with seizure-free surgical outcomes. The evaluation criterion in these studies is primarily the visual matching of the detected seizure foci with the true SOZ. The average accuracy of the presented studies in Table 1 is 61%, which is equivalent to a total of 152 concordant results out of 250 subjects who underwent surgery and were free from epileptic attacks.

In this study, a novel method is presented to localize the epileptic foci using fMRI data, that is easily implemented and avoids the previously mentioned disagreements. Our dataset included the fMRI data from 13 patients with temporal lobe epilepsy who were candidates for resection surgery. Ten patients had resection surgery with seizure-free outcomes after a follow-up of more than 12 months. The method presented in this paper is non-invasive in nature with no requirement of simultaneously recorded EEG signals. This mainly simplifies data acquisition, artifact removal, and implementation, in addition to avoiding the mentioned questions about the basis of EEG-fMRI studies by being independent of the spike detection process and the foci depth.

## 2 Materials and methods

**Figure 1:**
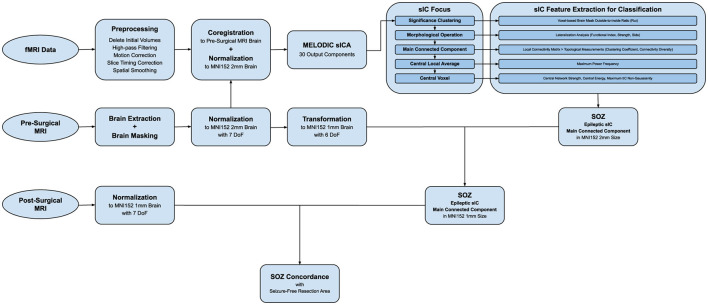
Diagram of the presented method algorithm.

The presented method in this study is based on ST-ICA on rs-fMRI data with a component-sorting procedure based on dominant power frequency, biophysical constraints, spatial lateralization, local connectivity, temporal energy, and functional non-Gaussianity. Figure 1 shows the diagram of the method presented in detail, including each step of the whole analysis algorithm from the raw data to the SOZ localization and concordance assessment.

### 2.1 Patients and data

This research was reviewed and approved by the Research Ethics Board (Institutional Review Board, IRB) of the Tehran University of Medical Sciences. Patients with severe cognitive impairment or other neurological diseases were excluded from the study. Patients whose cognitive impairments prevented participation in the full structural, diffusion, or functional MRI study were also excluded. All volunteers signed informed consent to participate in the study. Thirteen unilateral TLE subjects who had undergone resection surgery and had seizure-free (Engel I) outcomes after a 12-month follow-up were recruited. The dataset includes pre-surgical MRI and fMRI data for all patients along with post-surgical MRI images for 10 of them. All subjects were scanned using a 3-Tesla Siemens Magnetom Prisma MRI. Anatomical images were acquired for clinical diagnosis including transverse T1-weighted images with TR = 1,840 ms, TE = 3.47 ms, flip angle = 8°, matrix = 256 × 256, and slice thickness = 1.0 mm. The rs-fMRI were acquired in transverse planes covering the whole brain with 330 measurements. The parameters of the echo planar imaging sequence included TR = 3,000 ms, TE = 30 ms, flip angle = 90°, matrix = 640 × 640, slice thickness = 2.4 mm. For each subject, the duration of each fMRI measurement was ~16.5 min. All subjects were asked to keep their eyes closed during the rs-fMRI scanning process. Emphasis was placed on them not sleeping and confirmation was obtained after the scan that they had remained awake throughout. The clinical and electrophysiological characteristics of the patients are available in Table 2.

**Table 2:** Clinical and electrophysiological information of the patients.

**No**.	**Frequency**	**Onset (years)**	**Handedness**	**Semiology (salient features)**	**Ictal EEG (LTM)**	**Epileptogenic zone (LTM)**	**Irritative zone (LTM)**	**MRI (MTS)**	**Laterality**	**Outcome**
Sub001	1/m	28	R	Behavioral arrest with oral automatisms; verbalization (R)	Rhythmic alpha & theta activity R (T>F)	R (T)	R > L (F < T)	R	R	Engel I
Sub002	–	13	R	Bilateral limb automatisms	Rhythmic theta activity L=R (T)	L = R (F < T)	(R > L) (T)	R	R	Engel I
Sub003	7–12/w	13	R	Experiential aura; behavioral arrest; right limb dystonia (L)	Rhythmic theta activity L (T)	L (T)	R > L (T)	L	L	Engel I
Sub004	4/m	0.6	R	Behavioral arrest with staring; right limb dystonia; verbalization	Rhythmic delta activity R (T > F), L (T) Rhythmic theta activity R (T) Rhythmic delta activity L > R (T)	R (T)	R (T)	R	R	Engel I
Sub005	0.3–1/m	22	R	Left versive motion; left limb dystonia (R)	Rhythmic alpha activity R > L (T)	R (T)	R (T)	R	R	Engel I
Sub006	4/w	3	R	Staring with oral automatisms; right versive motion, right facial clonic activity (L)	Rhythmic theta activity L > R (T)	L (T)	–	L	L	Engel I
Sub007	1–4/w	19	R	Behavioral arrest; left limb automatism, right versive motion (L)	Rhythmic theta activity L > R (T)	L (T)	L (T)	L	L	Engel I
Sub008	–	–	–	Behavioral arrest with staring	–	R (T)	–	–	R	Engel I
Sub009	7–12/m	4	L	Experiential aura; behavioral arrest; Right Arm Dystonia	Rhythmic theta activities L > R (T)	L (T)	L (T)	L	L	Engel I
Sub010	2–3/w	2	R	Behavioral arrest with staring and oral automatism, spitting; left limb dystonia	Rhythmic theta R > L (T) Rhythmic delta R > L (T > F)	R (T)	(R > L) (T)	R > L	R	Engel I
Sub011	2–3/m	14	R	Behavioral arrest with staring	–	L (T)	–	L	L	Engel I
Sub012	1–4/m	11	R	Behavioral arrest with blinking and oral automatisms	Rhythmic theta activities L > R (T)	–	L	L	L	Engel I
Sub013	1–15/m	-	R	Behavioral arrest with blinking; left limb dystonia; ictal laughter (R)	Rhythmic alpha activity R > L (T)	R (T)	R (T)	R	R	Engel I

LTM, Long-Term Monitoring; MTS, Mesial Temporal Sclerosis.

### 2.2 Preprocessing

The fMRI data were preprocessed and analyzed using FSL (FMRIB Software Library, https://fsl.fmrib.ox.ac.uk/fsl) and Python. The first 10 volumes were discarded to ensure steady-state magnetization. A high-pass temporal filter with a cutoff of 100 s was applied to the fMRI data to eliminate the low-frequency drifts. Head motion correction was performed via a six-parameter rigid-body transformation based on the MCFLIRT algorithm. Temporal autocorrelations were corrected with an autoregressive model of order one. The data were spatially smoothed using a Gaussian filter with 6-mm full-width at half-maximum (FWHM) to increase the signal-to-noise ratio (SNR).

Functional images were first registered to the pre-surgical structural MRI. They were then normalized to the MNI152 brain template with a 2-mm voxel size using 7 degrees of freedom (DoF). For later concordance assessment, the full-skull post-surgical structural MRI was normalized to the non-brain-extracted MNI152 template with 1-mm voxel size using 7 DoF. A mutual-information cost function was used to retain the shape of the resected brain region. We also calculated the transformation from normalized pre-surgical structural MRI scan to the MNI152 brain template with 1-mm voxel size using 6 DoF. This allows the resulting SOZ to be mapped into high-resolution space for concordance assessment with the normalized post-surgical scan.

sICA was applied to fMRI data to explore and classify the candidate components associated with epilepsy (45). Thirty components with variance-normalized time courses were first computed for each patient (39). The voxel intensities of each sIC map were then converted to *Z*-scores to represent the spatial distribution of each component. Five levels of clustering were considered for feature extraction of each sIC: significance clustering required every cluster to have more than 10 contiguous voxels with *Z-*score >3.1; morphologically operated clusters were made after opening and closing with a 2-voxel disk; the main connected component was the connected cluster containing the sIC center, defined as the voxel with the maximum *Z-*score; the central local average was the averaged fMRI data in a 3-voxel neighborhood of the sIC center; and the central voxel was the fMRI data of the sIC center.

### 2.3 Feature extraction

For feature extraction, a set of potential functional biomarkers for epileptic focal activity were considered. A key innovation of our method is the extraction of a comprehensive set of features from each independent component (IC) of the rs-fMRI. These features serve as indicators to distinguish the epileptic ICs from non-epileptic ones. We extracted six features from each spatial IC identified by ICA, chosen based on known characteristics of epileptic foci and networks. This set is based on frequency features, biophysical constraints, spatial lateralization, local connectivity, temporal energy, and functional non-Gaussianity as follows.

#### 2.3.1 Biophysical constraints (voxel-based outside-to-inside ratio)

A spatial feature measuring the ratio of an IC's activity in peripheral/meningeal areas vs. core brain regions. According to the expectation that neurological activities being generated by neurons residing within the gray matter of the cortex, those components confounded with external sources of artifacts should have been excluded. Therefore epileptic ICs reflecting true neuronal activity should be largely contained within the brain parenchyma. A high outside-to-inside ratio might indicate that an IC represents noise, motion, or physiological artifacts (e.g., head movement or cardiovascular pulsation affecting edges of the brain). We apply this constraint in order to exclude ICs that do not represent genuine neural signals. In practice, all candidate ICs must have a low outside-to-inside ratio (i.e., predominantly intracerebral) to be considered a potential SOZ network. This feature therefore acts as an initial filter to improve specificity and reduce false positives from non-neuronal signals. To this end, fMRI voxels outside of the brain, which are caused by noise, were retained before performing sICA. Because they were included in the noisy components, they were used to identify and reject those noisy signals within the brain that have a statistical correlation with them. The index *R*_*o*/*i*_ was calculated as follows to discriminate the cortical components from noisy ones.


(1) $$\begin{array}{l} R_{\frac{o}{i}}=\frac{number\;of\;voxels\;outside\;the\;brain}{number\;of\;voxels\;inside\;the\;brain}\;. \end{array}$$

#### 2.3.2 Maximum power frequency

Based on the expected temporal structure in each component to be neurophysiologically meaningful and normal (46), the dominant frequency of each sIC's central local average was considered in our feature set. The averaged fMRI data were calculated in a 3-voxel neighborhood around the maximum *Z-*score voxel as the sIC center. A periodogram was then used to find the dominant frequency. Epileptic regions might exhibit distinctive spectral power profiles, such as enhanced power at specific slow frequencies, possibly reflecting neurovascular coupling to epileptic activity. By identifying the dominant frequency component of each IC, we assess whether an IC's temporal activity has an unusual concentration of power that might signal epileptic activity. This feature helps discriminate physiological networks from potentially pathological ones. For unprocessed fMRI data, a dominant frequency above 0.1 Hz reflects aliasing from respiration and cardiac artifacts, while lower than 0.01 Hz is due to scanner susceptibility artifacts. Therefore, the components with dominant power outside of the frequency range of 0.01–0.1 Hz should be excluded from further analyses.

#### 2.3.3 Functional lateralization index

This measure quantifies how asymmetrically the IC's spatial map is distributed between hemispheres. Resting-state brain activities are assumed to be relatively symmetric, whereas epileptic activity tends to lateralize to one hemisphere (47). Under this assumption, epileptic components should show less relative symmetry about the anterior commissure–posterior commissure (ACPC) plane. Conversely, ICs corresponding to normal resting-state networks are usually symmetric or present in both hemispheres. This is under the assumption that the right number for extracted components in order to prevent a split in resting-state symmetric ones. We flattened the mirroring voxels of each component about the ACPC plane into two one-dimensional arrays. Pearson's correlation coefficient was then computed between these arrays to quantify symmetry. The functional lateralization index was defined for each component as follows:


(2) $$LI=1-\left|\frac{\sum_{i=1}^{n}\left(x_{i}^{L}-x^{L}\right)\left(x_{i}^{R}-x^{R}\right)}{\sqrt{\sum_{i=1}^{n}{\left(x_{i}^{L}-x^{L}\right)}^{2}\sum_{i=1}^{n}{\left(x_{i}^{R}-x^{R}\right)}^{2}}}\right|\;.$$

#### 2.3.4 Lateralization strength

Following the functional lateralization index, lateralization strength is defined as a measure of significant binary clusters of each component. After thresholding the component by retaining clusters with *Z-*score >3.1 and more than 10 contiguous voxels, lateralization strength was defined on non-mirrored binary voxels about the ACPC plane as follows:


(3) $$LS=\frac{\left|\sum X_{L}-\sum X_{R}\right|}{X_{L}+X_{R}}\;.$$

#### 2.3.5 Local clustering coefficient

Local connectivity is a measure of how strongly the voxels within the IC's region connect with each other relative to the rest of the brain. We derive this from the correlation among the time-series of voxels within the spatial IC main cluster. To this end, we applied morphological opening and closing operations with a 2-voxel disk. The main connected component was chosen among the connected clusters, namely the cluster containing of the maximum *Z-*score voxel as the sIC center. The local connectivity matrix was calculated from the fMRI data inside the main cluster of each sIC for later topological measurements. The local clustering coefficient provides a measure of the level of cliquishness or local interconnectedness of a network (13, 48). This feature complements the spatial and spectral features by adding a network perspective. This measure is defined for the local connectivity of each sIC as the ratio of the sum of existing to the sum of possible connections in the subnetwork:


(4) $$\begin{array}{l} CC=\frac{\underset{i\neq j}{\sum }\left(r_{ij}\right)}{N\left(N-1\right)}\;. \end{array}$$

#### 2.3.6 Local connectivity diversity

Local connectivity diversity provides a measure of the heterogeneity of the local network connectivity in each IC. This measure is defined as the unbiased sample variance of all pairwise correlations within the sIC subnetwork:


(5) $$CD=\frac{1}{N-1}\sum_{j\neq i}{\left(r_{ij}-r\right)}^{2}.$$

#### 2.3.7 Central network strength

Central network strength provides a measure of the average level of connectivity between the sIC center and the rest of the sIC voxels.


(6) $$\begin{array}{l} NS=\frac{1}{N-1}\;\underset{i\neq c}{\sum }r_{ic}\;where\;c:\;sIC\;center\;. \end{array}$$

#### 2.3.8 Central energy

Epileptic activity (even interictal) can cause sporadic surges in the BOLD signal, leading to higher variance over time. Components capturing these fluctuations would have a higher temporal energy. We use this feature to detect ICs that are particularly “active” or erratic throughout the scan. The epileptic IC might stand out by having a higher signal variance (beyond normal resting fluctuations) due to interictal epileptiform events or ongoing baseline dysfunction in that region. However, we interpret this feature with caution, as high variance can also come from motion; hence, it is used in conjunction with the biophysical constraint feature to ensure we are measuring neural-derived energy. Therefore, the central energy is calculated from the energy of the fMRI data at the sIC center as a feature for candidate sorting.


(7) $$\begin{array}{l} CE=\underset{t}{\sum }{x_{c}\left(t\right)}^{2}\;where\;c:\;sIC\;center\;. \end{array}$$

#### 2.3.9 Maximum tIC non-Gaussianity

Another measure for candidate sorting is the maximum non-Gaussianity among the temporal sources of activity in each sIC subregion. This is a measure of how non-Gaussian the distribution of the IC's voxel intensities or time series is, which can be related to the presence of structured, non-random activity. ICA itself finds components by maximizing non-Gaussianity (via kurtosis or negentropy); here, we specifically evaluate whether the extracted IC's spatial or temporal patterns deviate significantly from a normal (Gaussian) distribution. An epileptic network might produce highly kurtotic temporal activity (e.g., brief spikes in activity) as opposed to smoother, diffuse components (like physiological networks), which are expected to be Gaussian in their activation spread (42). To this end, the time series of independent sources in the activation region of each candidate component were separated by tICA, and the temporal signal with the largest absolute value of Gaussian deviation (kurtosis) was considered the representative of the epileptic temporal activity. The kurtosis of *y* is defined as follows (49):


(8) $$\begin{array}{l} Kurtosis\;\left(y\right)=E\left(y^{4}\right)-3\left(E{\left(y^{2}\right)}^{2}\right)\;, \end{array}$$

where *E*(*y*) is the expected value of *y*. After identifying the epileptic temporal signal from each candidate component, the final epileptic foci can also be localized using seed-based functional connectivity analysis. The aforementioned feature sets for the 30 sICs of the 10 subjects are provided in the Appendix. These features are to be classified as focal and non-focal for localization of epileptic foci.

### 2.4 Component classification

Each of these features captures a different aspect of the data, and **our** classification approach considered all features together to identify the most likely epileptic IC. In our procedure, the feature set was classified using a meaningful analytic thresholding approach. We applied a cut-off of one standard deviation (SD) below the mean of all sICs and subjects toward the expected direction of the feature. Specifically, features were evaluated based on their maximum power frequency (between 0.01 and 0.1 Hz), voxel-based outside-to-inside ratio (less than the threshold), functional lateralization index (greater than the threshold), lateralization strength (greater than the threshold), local clustering coefficient (less than the threshold), local connectivity diversity (greater than the threshold), central network strength (less than the threshold), central energy (maximum among candidates), and maximum tIC non-Gaussianity (maximum among candidates). The last two features were prioritized among the candidates that passed the initial thresholding procedure in each patient. Therefore, one sIC was chosen through this procedure for each patient as the epileptic candidate. We found that this multi-criterion sorting was effective. in most of the patients, one IC emerged that fit the epileptic profile, and whose spatial map corresponded closely with the known epileptic focus, as later confirmed by resection location and outcome.

### 2.5 Feature importance

To ensure that our feature-based classification was robust, we performed analyses to gauge the contribution of each feature and the risk of overfitting given our sample size. We observed that certain features were especially influential. For instance, the outside-to-inside ratio was crucial for filtering out non-neural components; without this filter, many false ICs (e.g., representing motion or CSF pulsation) could have been misidentified as “epileptic” due to high variance. The dominant frequency and spatial lateralization features were highly consistent indicators—in all patients, the SOZ-IC had a uniquely high lateralization (matching the side of surgery) and a dominant frequency near ~0.01–0.04 Hz, distinguishing it from other components. The local connectivity feature further boosted confidence in the chosen IC: in most cases, the epileptic IC showed a diverse local connectivity with higher energy and non-Gaussianity. We did not find a single feature that alone perfectly separated the SOZ-IC in every case; instead, it was the combination that proved reliable. This underscores why a fuzzy or multi-feature classifier (as we discuss later) is appropriate for such complex problem in case of having adequate data. We also performed a retrospective feature importance check: if we removed one feature at a time from the decision process, the accuracy of localization would drop. This suggests that all feature categories we included carry useful, non-redundant information for SOZ identification.

Given our relatively small sample (*N* = 13), there is a possibility that the feature thresholds or weights we implicitly used could be overfit to our dataset. We mitigated this risk by using consistent criteria derived from domain knowledge rather than tuning to each patient or optimizing on outcomes. Additionally, we validated our approach by checking concordance with surgical resection and outcomes, rather than only an internal cross-validation. The fact that the fMRI-predicted focus matched the surgically removed tissue leading to seizure freedom provides an independent validation of the feature selection logic. Nonetheless, we acknowledge that with such a limited cohort there remains a risk that some features or their cutoff values might not generalize to all epilepsy cases. We address this in the Limitations and Future Directions section. Overall, our feature analysis confirms that each category of feature contributes to identifying the SOZ, and using them in concert was key to the method's success. Careful cross-validation on larger cohorts in the future will be important to ensure the features are generalizable and model isn't overfitting.

## 3 Results

A total of thirteen patients were recruited in this study. All patients had been diagnosed as surgical candidates with temporal lobe epilepsy. They underwent resection surgery with seizure-free outcomes after at least a 12-month follow-up. Ten subjects who had post-surgical MRI scans were included for the concordance assessment with the localization results. After preprocessing, sICA was applied to each fMRI data and thirty spatially independent components with variance-normalized time courses were extracted. The feature set was obtained for all 300 components from all patients to calculate the sample means and standard deviations. We then defined the classification threshold for each feature based on those statistics. The epileptic sIC was extracted for each subject based on the previously described protocol. Table 3 shows the feature set for the epileptic sIC across all the subjects.

**Table 3:** The feature set of the epileptic sICs.

**Subject ID**	**sIC number**	**sIC center**	**R_o/i**	**Max power frequency**	**Lateralization index**	**Lateralization strength**	**Lateralization side**	**Central network**	**Clustering coefficient**	**Connectivity diversity**	**Central energy**	**Max tIC non-gaussianity**
1	16	(60.0, 22.0, 12.0)	0.04	0.036	0.756	0.994	Right	0.482	0.387	0.043	95,325,948,678.60340	315.003133545483
2	17	(62.0, −8.0, 22.0)	0.018	0.025	0.51	0.411	Right	0.56	0.475	0.049	32,458,319,850.63030	315.003133729914
3	13	(−64.0, −48.0, 26.0)	0.092	0.017	0.637	0.588	Left	0.348	0.231	0.053	37,430,046,193.99810	315.003134102643
4	15	(38.0, −52.0, 64.0)	0.061	0.02	0.718	1	Right	0.237	0.213	0.104	110,812,658,071.97300	315.003133634571
5	27	(58.0, −56.0, 32.0)	0.156	0.018	0.705	0.543	Right	0.433	0.339	0.074	34,834,436,821.58160	315.003132862966
6	17	(−54.0, 26.0, −6.0)	0.189	0.027	0.829	0.944	Left	0.363	0.266	0.049	42,803,728,111.47780	315.003133696123
7	26	(−50.0, −44.0, 56.0)	0.184	0.016	0.9	0.976	Left	0.336	0.238	0.08	409,992,669,118.26000	315.003130300942
8	4	(58.0, 16.0, −2.0)	0.217	0.034	0.521	0.578	Right	0.402	0.261	0.059	83,131,683,704.61460	315.003130164952
9	15	(−22.0, −44.0, 82.0)	0.295	0.024	0.742	0.604	Left	0.08	0.131	0.089	12,609,534.58141	315.003126433931
10	10	(64.0, 20.0, 8.0)	0.115	0.027	0.407	0.805	Right	0.41	0.417	0.066	8,724,295,223.41954	315.003133076034

**Figure 2:**
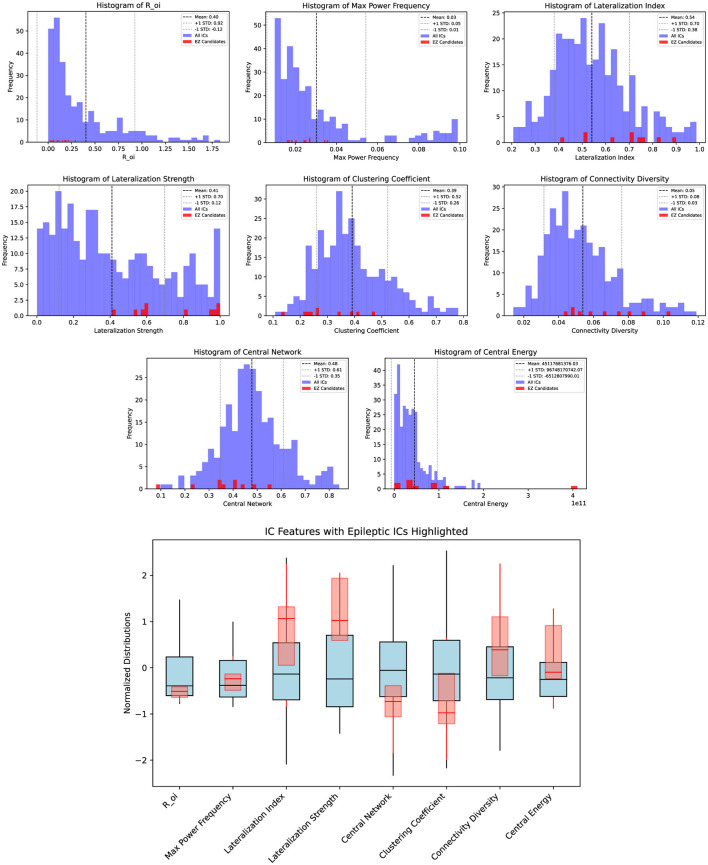
Histogram of features among all sICs and subjects with the epileptic sICs being highlighted (**Top**) and the boxplot of the feature set with a normalized range among all sICs and subjects with the epileptic sICs being highlighted (**Bottom**).

The histograms of each feature and the integral boxplot of the feature set with a normalized range among all 300 components are shown in Figure 2 with those of epileptic ICs being highlighted on each.

**Figure 3:**
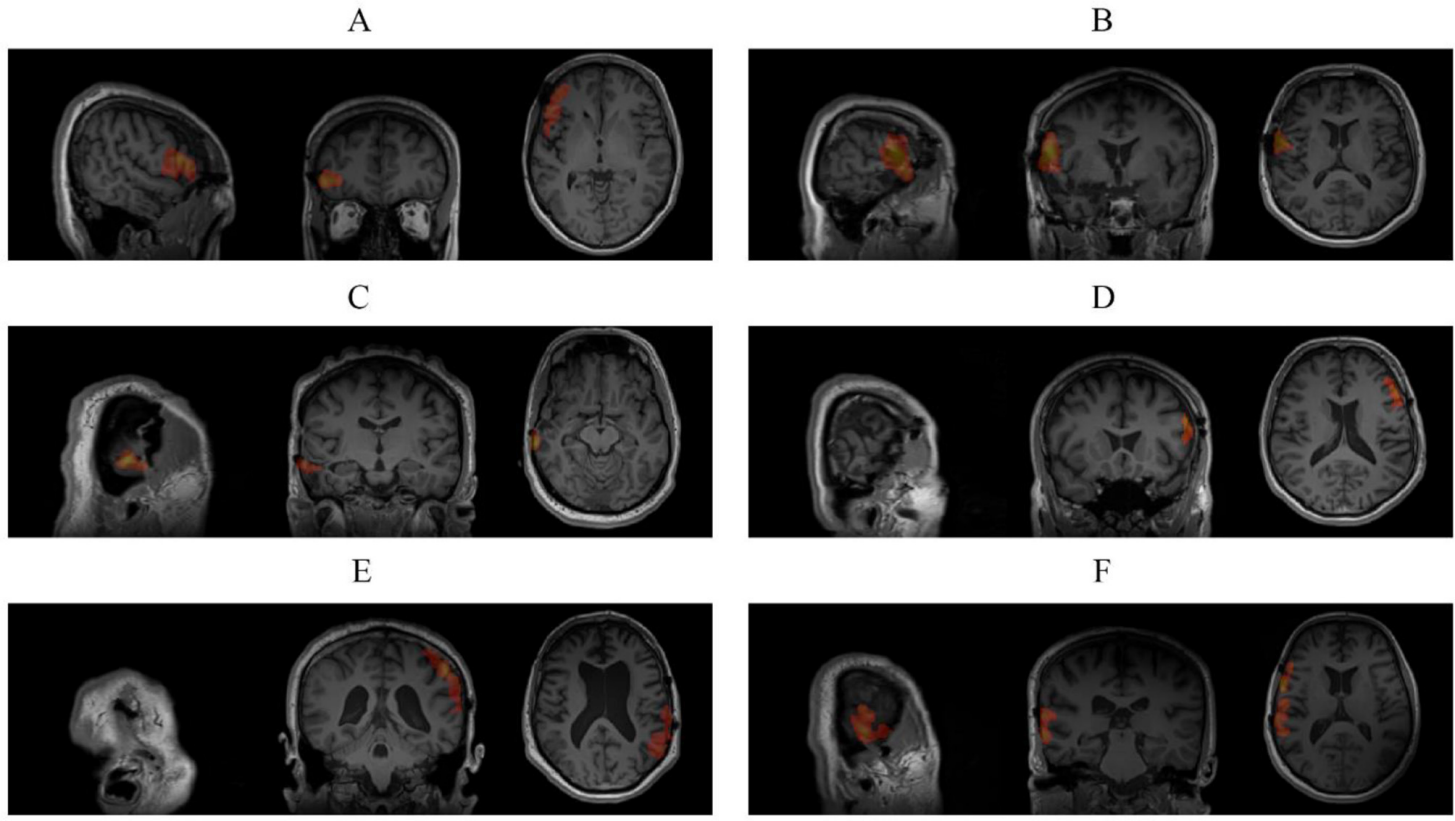
The post-surgical MRI with overlaid localized SOZ cluster on resection area using the presented method: **(A)** subject 1 with behavioral arrest with oral automatisms and verbalization showing rhythmic alpha and theta activity at right temporal lobe (T4>F8) in ictal EEG; **(B)** subject 2 with bilateral limb automatisms showing rhythmic theta activity at both temporal lobes (T>F) but right irritative zone in ictal EEG; **(C)** subject 5 with left versive motion and left limb dystonia showing rhythmic alpha activity at right temporal lobe (T4>T3) in ictal EEG; **(D)** subject 6 with staring with oral automatisms, right versive motion, and right facial clonic activity showing rhythmic theta activity at left temporal lobe (T3>T4) in ictal EEG; **(E)** subject 7 with behavioral arrest, left limb automatism, and right versive motion showing rhythmic theta activity at left temporal lobe (T3>T4) in ictal EEG; **(F)** subject 8 with behavioral arrest with staring.

The selected components identified as epileptic tend to be inside the brain, highly lateralized, low in their central network strength and local clustering coefficient, and high in their local connectivity diversity, central energy, and functional non-Gaussianity. After classification, the epileptic sIC was clustered into its connected components with *Z-*score >3.1 and more than 10 contiguous voxels, smoothed by morphological opening and closing with a 2-voxel disk, and reduced to its main connected component containing the maximum *Z-*score voxel as the resulting SOZ. The SOZs were overlaid on the corresponding post-surgical MRI scan and divided into three levels of concordance. Fully concordant results were spatially aligned with the surgical resection. Partially concordant results were in the same lobe without overlapping the resection area, while discordant results had clusters outside the resection lobe. The presented method yielded six fully concordant results with precise localization, three partially concordant results with correct lateralization and lobe yet no overlap with the resection area, and one discordant result outside the resection lobe. Figure 3 shows the post-surgical images of six patients with fully concordant results overlaid.

## 4 Discussion

Localization of epileptic foci has become an important step in presurgical evaluation providing a primary perspective of the region of interest in surgery (50). Although ictal studies and invasive approaches such as iEEG are known as the gold standard for this purpose, they demand substantial resources (14). Meanwhile, interictal studies and non-invasive methods could address these challenges if they allow rapid, comprehensive detection of interictal discharges along with a dependable protocol for localizing seizure sources (13). Multi-modal non-invasive data recordings, especially simultaneous EEG-fMRI have attracted a great deal of attention over the past few years. The idea of combining the temporal resolution of EEG with the spatial resolution of fMRI sounds promising for precisely localize a source of abnormal activity in the brain (19, 28, 29).

However, not all studies in the literature support this idea (31, 32). EEG and fMRI measure different phenomena related to epilepsy possibly occurring at different time scales. Consequently, the relationship between BOLD signal and LFP is not strictly linear and can vary with multiple factors. Functional connectivity measured by BOLD and EEG signals often show relatively weak correlation, and EEG spikes can lead to increased, decreased, or unchanged BOLD signals (30). Moreover, EEG-fMRI methods typically require frequent, well-expressed spikes on the scalp EEG. This becomes a limitation when the epileptic source lies in deep brain structures (12, 36).

The analysis of interictal scalp EEG alone based on ICA and ESI will lead to poor localization results (8). Direct analysis of interictal rs-fMRI data, on the other hand, has been shown to provide valid information about epileptic sources. Various tools exist to study abnormal activity sources in brain volume from rs-fMRI data. They apply selection criteria to the components extracted by sICA. Temporal analysis is another useful tool to find and confirm a subregion responsible for seizure generation within the epileptogenic zone. Connectivity analysis reveals functionally integrated relationships within clusters and between distant brain regions. This approach helps identify areas significantly connected to the SOZ and the propagation pathways of epileptic activity. Testing and comparing these methods on patient cohorts has always provided valuable insights. Such work guides the development of optimal presurgical localization procedures and the proposal of novel techniques.

Earlier studies have demonstrated that rs-fMRI can detect abnormalities related to the SOZ. For example, Lee et al. (13) used voxel-wise intrinsic connectivity contrast (ICC) maps to localize epileptogenic zones and reported ~72% concordance with intracranial EEG-defined SOZ in 29 patients. Notably, their fMRI localization was more successful in patients who went on to have good surgical outcomes, especially in TLE cases, underscoring that fMRI markers of the SOZ tend to align with clinically relevant targets. A recent meta-analysis further confirmed that rs-fMRI can contribute to SOZ identification with high sensitivity (~91% on average) (50). However, that meta-analysis also reported low specificity (many false positives) for fMRI alone (50), reflecting a tendency of fMRI to highlight broad epileptic networks beyond the true focus. These prior works establish that while rs-fMRI is a promising non-invasive tool, purely connectivity-based or univariate approaches may flag widespread network changes, making it challenging to pinpoint the exact focus without additional information.

In contrast to most previous fMRI studies that relied on either seed-based functional connectivity or EEG-fMRI spike correlations, our approach introduces a spatio-temporal ICA component classification tailored to isolate the putative epileptic network component in each patient. This method does not require simultaneous EEG or the occurrence of interictal spikes during the scan, which is a key advancement. For instance, methods integrating EEG with fMRI have shown efficacy in identifying SOZ (51). While effective, those require EEG recordings and, in most studies, detectable epileptic discharges during fMRI. Our technique operates solely on rs-fMRI data, using features of the independent components to automatically select the component corresponding to the SOZ.

Compared to related studies, such as Lee et al. (13) who found decreased intra-hemispheric connectivity in the SOZ region, our approach not only considers connectivity (through a local connectivity feature) but also incorporates multiple other biomarkers (frequency content, spatial distribution, etc.) in a unified classification framework. This multi-feature approach likely contributes to improved specificity—instead of labeling all areas of altered connectivity as SOZ, we pinpoint the component that simultaneously meets several epileptic criteria. Moreover, unlike group analyses of connectivity that demonstrate statistical differences between patients and controls (52), our method provides single-subject SOZ localization, which is directly usable in presurgical planning. Recent works by Kamboj et al. (53, 54) took a different approach, employing deep learning on rs-fMRI combined with expert input to identify SOZ in pediatric epilepsy. Their hybrid model of expert knowledge and AI achieved high sensitivity (~90%) and reduced the expert's workload significantly (53, 54). Our work is complementary to such efforts: rather than using a black-box deep network, we use an explainable feature-based classification. This means our results can be interpreted in terms of neurophysiological features (e.g., dominant frequency or spatial extent), which could provide insights into the nature of the detected epileptic network.

In summary, our study advances the field by demonstrating that a purely fMRI-based, feature-driven method can localize the SOZ with accuracy comparable to EEG-informed or deep learning methods while being fully non-invasive and transparent. The high concordance of our localized foci with surgical resection zones (and the resulting seizure freedom) underscores the practical clinical value of this advancement. Our successful localization of the SOZ using interictal fMRI further supports the notion that the epileptic network's imprint on brain activity (even outside seizures) can be detected and used clinically. In comparison to prior research, therefore, this study provides a novel and effective framework for single-subject SOZ localization and bridges the gap between network-level observations and actionable focal targets.

## 5 Conclusion

In this study, a novel method has been presented to localize epileptic foci based on the analysis of rs-fMRI data alone with no requirement for simultaneously recorded EEG signals. The required functional data for this method are more accessible, cost-efficient, and easier to record compared to other methods based on EEG-fMRI, ictal Video-EEG, MEG, and iEEG. There are fewer assumptions when using rs-fMRI data about the biological features of epilepsy. Moreover, localizing based on rs-fMRI data alone makes the method independent of the depth of epileptic foci and alleviates concerns about the occurrence and detection of epileptic spikes. This method aimed to achieve high spatial accuracy in localizing epileptic foci from interictal data while retaining the reliability of results for clinical usage. After evaluation on a group of patients, the presented method showed promising results compared with the literature highlighting valuable features that serve as SOZ functional biomarkers.

The presented method is based on spatio-temporal ICA component classification for localizing the seizure onset zone using only rs-fMRI data. Applied on 13 TLE patients, this approach achieved high concordance with surgical resection sites, indicating that our non-invasive method successfully identified the epileptic focus in each case. All patients in our study had Engel I outcomes at 12 months, meaning they were completely seizure-free 1 year after surgery. This uniform positive outcome strongly supports the validity of the SOZ localizations provided by our fMRI analysis. In practical terms, the zones identified by our method corresponded to the tissue that, when resected, led to sustained seizure freedom. Although the data are reassuring after the 12-month follow-up, we will continue to track long-term outcomes for any additional patients studied with this approach. Any recurrence or breakthrough seizures in the long term will be analyzed to see if they correlate with aspects of the fMRI data (for example, involvement of network nodes not resected).

This method advances the state-of-the-art by removing the dependency on concurrent EEG or actual seizures during scanning, instead relying on multi-faceted features of interictal brain activity. We have shown that features such as low-frequency BOLD power, spatial focus, and network connectivity can serve as reliable biomarkers of epileptic foci (55). By comparing our results with prior studies, we conclude that our technique offers comparable accuracy to more resource-intensive approaches, and importantly, it provides interpretable results that align with clinical ground truth (surgical outcome).

In conclusion, this study provides a proof-of-concept that rs-fMRI, analyzed with sophisticated ICA and feature-classification techniques, can localize epileptic foci in a manner that corresponds with surgical success. The work pushes beyond previous research by eliminating the need for concurrent EEG or ictal data, instead leveraging inherent patterns in interictal brain activity. Our findings contribute to the growing paradigm of network-based epilepsy diagnostics, and we believe they lay a foundation for non-invasive, data-driven tools that complement clinical expertise.

## 6 Limitations and future directions

While our results are promising, we are cognizant of several limitations in the current study and outline future directions to enhance the approach.

### 6.1 fMRI—only localization and mislocalization risk

As noted in prior studies, rs-fMRI may show high sensitivity but low specificity in detecting epileptic regions (50)—meaning it might highlight a broad epileptic network rather than the precise epileptogenic zone. In our study, we addressed this by using stringent feature-based criteria to hone in on the likely focus, but there remains a risk that the identified component, while concordant with the resection area in our cases, could in other patients represent a prominent part of the epileptic network rather than the actual minimal EZ. We did observe that in a few patients, the selected IC's cluster extended into regions adjacent to the resected zone (though still within the same lobe). In clinical terms, such an outcome is still useful (since it localizes to the correct lobe), but finer precision is needed to delineate the exact EZ. To mitigate mislocalization, a clear future direction is to adopt a multimodal imaging approach. Combining fMRI with EEG can provide timing information to distinguish the true onset zone from propagation. Indeed, simultaneous EEG-fMRI has been shown to improve specificity by relating BOLD changes to actual epileptic discharges (51). Even if simultaneous EEG is not available, other modalities like MEG, SPECT, or PET could be integrated into a multimodal classifier to verify the fMRI-driven predictions.

### 6.2 Advanced classification methods

Another avenue to enhance localization accuracy is to employ advanced machine learning models that can handle complex multivariate patterns. Our current method uses a rule-based classification of ICs, which benefits from explainability. A machine learning model trained on a larger dataset could potentially learn subtle feature combinations or higher-dimensional representations that distinguish epileptic networks more reliably. Recent studies illustrate this potential: deep learning models applied to rs-fMRI of epileptic patients have shown success in detecting SOZ-related patterns, especially when combined with expert knowledge (53). In our context, a supervised learning approach could be used if sufficient training cases are available—the features we defined could serve as inputs to a classifier, such as a support vector machine or random forest, which could then output the probability of each IC being the SOZ. However, such models require larger datasets to avoid overfitting and to capture the variability across different epilepsy subtypes. We believe our feature set provides a strong starting point and could reduce the dimensionality burden for a learning model, although further validation and model training on multi-center data would be necessary. The accuracy of this classification could also be improved by adopting an approach based on fuzzy similarity, which would allow for handling uncertainty and variability in the extracted features, providing a more nuanced categorization and reducing the risk of misclassification. This approach would enable assigning a degree of membership to different categories rather than a strict distinction, making the method more flexible and robust against individual patient variations. Fuzzy similarity has been used before to localize and classify defects in materials, treating the problem as one of comparing signals to reference templates (56).

### 6.3 Small sample size and generalizability

A clear limitation of this study is the small sample size: we analyzed 13 patients, all with unilateral temporal lobe epilepsy and favorable post-surgical outcomes. While this homogeneity helped demonstrate proof-of-concept under ideal conditions (all had well-localized foci and Engel I outcomes), it may have biased the results. The method's performance in more heterogeneous or larger populations remains untested. We plan to initiate multi-center collaborations to apply our pipeline to a larger cohort of patients. A multi-center study would not only increase the sample size but also incorporate diversity in scanner hardware, epilepsy types, and clinical practices, which would test the robustness and generalizability of our approach. Such collaboration could involve prospectively collecting rs-fMRI from new patients and retrospectively applying our method to existing fMRI datasets from other epilepsy surgery centers. If successful, a multi-center validation demonstrating consistent localization accuracy would strongly support the clinical utility of the method.

## Data Availability

The dataset used in this study will be made available upon request to the authors.
