# Supplementary material for: Spatio-temporal independent component classification for localization of seizure onset zone
Source: Front Neurol. 2025 Jun 6;16:1515484. doi: 10.3389/fneur.2025.1515484 (PMC12180306; doi:10.3389/fneur.2025.1515484)
Supplement: Supplementary file 1 [file Table_1.docx]

Table 1. The feature set of 30 sICs for subject 1.

| Subject ID | sIC Number | sIC Center | R_o/i | Max Power Frequency | Lateralization Index | Lateralization Strength | Lateralization Side | Central Network | Clustering Coefficient | Connectivity Diversity | Central Energy | Max tIC  Non-Gaussianity |
| --- | --- | --- | --- | --- | --- | --- | --- | --- | --- | --- | --- | --- |
| 1 | 1 | (-16.0, 60.0, 18.0) | 0.042 | 0.05 | 0.262 | 0.171 | Left | 0.589 | 0.487 | 0.043 | 218223660429.39300 | 315.003132341517 |
| 1 | 2 | (2.0, -18.0, 68.0) | 0.008 | 0.024 | 0.379 | 0.28 | Right | 0.517 | 0.4 | 0.047 | 83677801822.67600 | 315.003131776078 |
| 1 | 3 | (-8.0, -96.0, -22.0) | 0.332 | 0.032 | 0.462 | 0.206 | Left | 0.55 | 0.414 | 0.053 | 18227473709.10360 | 315.003129539823 |
| 1 | 4 | (-40.0, -56.0, 56.0) | 0.055 | 0.024 | 0.625 | 0.699 | Left | 0.474 | 0.352 | 0.059 | 138077085622.43800 | 315.003128138078 |
| 1 | 5 | (-2.0, -88.0, 8.0) | 0.023 | 0.024 | 0.424 | 0.009 | Left | 0.622 | 0.443 | 0.052 | 51103927948.11310 | 315.003130339340 |
| 1 | 6 | (-34.0, -20.0, 64.0) | 0.028 | 0.024 | 0.364 | 0.108 | Right | 0.349 | 0.311 | 0.053 | 100595732661.69900 | 315.003132250685 |
| 1 | 7 | (-44.0, -74.0, 36.0) | 0.038 | 0.025 | 0.46 | 0.695 | Left | 0.404 | 0.275 | 0.057 | 70672113626.86750 | 315.003130826625 |
| 1 | 8 | (56.0, -64.0, 16.0) | 0.018 | 0.016 | 0.526 | 0.827 | Right | 0.346 | 0.242 | 0.063 | 81589355303.03070 | 315.003133618806 |
| 1 | 9 | (8.0, 30.0, 54.0) | 0.16 | 0.023 | 0.585 | 0.039 | Right | 0.435 | 0.34 | 0.045 | 71983514901.79310 | 315.003131977752 |
| 1 | 10 | (54.0, -44.0, 56.0) | 0.05 | 0.024 | 0.515 | 0.85 | Right | 0.314 | 0.244 | 0.072 | 258889984436.92900 | 315.003131781309 |
| 1 | 11 | (34.0, -84.0, 18.0) | 0.006 | 0.016 | 0.327 | 0.007 | Right | 0.381 | 0.24 | 0.076 | 97407799561.83400 | 315.003130944433 |
| 1 | 12 | (-56.0, -50.0, -22.0) | 0.396 | 0.015 | 0.882 | 0.81 | Left | 0.603 | 0.538 | 0.023 | 22342385200.90610 | 315.003130105648 |
| 1 | 13 | (8.0, 26.0, 58.0) | 0.193 | 0.023 | 0.516 | 0.394 | Right | 0.63 | 0.588 | 0.047 | 13009657743.40870 | 266.444834164436 |
| 1 | 14 | (24.0, -84.0, 42.0) | 0.108 | 0.024 | 0.424 | 0.105 | Left | 0.395 | 0.349 | 0.056 | 47409015703.38900 | 315.003131430749 |
| 1 | 15 | (62.0, -8.0, 34.0) | 0.025 | 0.011 | 0.494 | 0.829 | Right | 0.481 | 0.388 | 0.061 | 91049947220.77520 | 315.003129055849 |
| 1 | 16 | (60.0, 22.0, 12.0) | 0.04 | 0.036 | 0.756 | 0.994 | Right | 0.482 | 0.387 | 0.043 | 95325948678.60340 | 315.003133545483 |
| 1 | 17 | (-34.0, -94.0, -6.0) | 0.11 | 0.035 | 0.247 | 0.027 | Left | 0.464 | 0.299 | 0.074 | 79534801517.19230 | 315.003131914568 |
| 1 | 18 | (18.0, 8.0, -26.0) | 0.071 | 0.024 | 0.499 | 0.678 | Right | 0.489 | 0.473 | 0.033 | 12164017191.17300 | 315.003122412802 |
| 1 | 19 | (-38.0, -30.0, -30.0) | 0.321 | 0.015 | 0.836 | 0.92 | Left | 0.57 | 0.471 | 0.033 | 4406691689.97183 | 315.003131470809 |
| 1 | 20 | (50.0, -62.0, -44.0) | 0.111 | 0.032 | 0.973 | 0.977 | Right | 0.481 | 0.513 | 0.029 | 21472451794.96580 | 315.003130923913 |
| 1 | 21 | (-62.0, 12.0, 10.0) | 0.007 | 0.032 | 0.588 | 0.845 | Left | 0.476 | 0.384 | 0.033 | 80263372145.95990 | 315.003133079224 |
| 1 | 22 | (10.0, -38.0, -68.0) | 0.115 | 0.094 | 0.594 | 0.1 | Right | 0.566 | 0.526 | 0.029 | 12235076644.42330 | 315.003063741491 |
| 1 | 23 | (52.0, 22.0, -22.0) | 0.11 | 0.025 | 0.45 | 0.585 | Right | 0.275 | 0.271 | 0.059 | 57939537814.84450 | 315.003131906222 |
| 1 | 24 | (10.0, -106.0, -18.0) | 0.531 | 0.015 | 0.594 | 0.462 | Right | 0.764 | 0.702 | 0.021 | 18730431968.64210 | 315.002936066640 |
| 1 | 25 | (56.0, 16.0, 28.0) | 0.061 | 0.028 | 0.566 | 0.859 | Right | 0.28 | 0.229 | 0.066 | 102417786732.07300 | 315.003132925178 |
| 1 | 26 | (-30.0, 34.0, -30.0) | 0.386 | 0.024 | 0.771 | 0.54 | Right | 0.792 | 0.675 | 0.034 | 11069402757.71770 | 245.305364306678 |
| 1 | 27 | (-34.0, -42.0, -38.0) | 0.199 | 0.011 | 0.767 | 0.287 | Left | 0.791 | 0.687 | 0.03 | 11640163972.92820 | 50.697834951354 |
| 1 | 28 | (-44.0, 6.0, -18.0) | 0.019 | 0.024 | 0.474 | 0.638 | Right | 0.552 | 0.49 | 0.036 | 40661298974.46490 | 315.003127680335 |
| 1 | 29 | (30.0, 48.0, -24.0) | 0.339 | 0.015 | 0.973 | 0.096 | Right | 0.505 | 0.367 | 0.037 | 10200234864.72010 | 315.003123088942 |
| 1 | 30 | (-16.0, -16.0, -46.0) | 0.129 | 0.093 | 0.609 | 0.299 | Right | 0.518 | 0.494 | 0.04 | 1525150601.62384 | 315.003102502021 |

Table 2. The feature set of 30 sICs for subject 2.

| Subject ID | sIC Number | sIC Center | R_o/i | Max Power Frequency | Lateralization Index | Lateralization Strength | Lateralization Side | Central Network | Clustering Coefficient | Connectivity Diversity | Central Energy | Max tIC  Non-Gaussianity |
| --- | --- | --- | --- | --- | --- | --- | --- | --- | --- | --- | --- | --- |
| 2 | 1 | (-2.0, -50.0, 60.0) | 0.746 | 0.039 | 0.464 | 0.384 | Left | 0.751 | 0.68 | 0.021 | 22723058873.82870 | 315.003125537979 |
| 2 | 2 | (-58.0, 10.0, -4.0) | 0.142 | 0.029 | 0.425 | 0.281 | Right | 0.644 | 0.555 | 0.06 | 29681855096.31260 | 315.003123738863 |
| 2 | 3 | (0.0, -80.0, 12.0) | 0.074 | 0.039 | 0.213 | 0.556 | Left | 0.545 | 0.367 | 0.066 | 39900603450.76830 | 315.003131587168 |
| 2 | 4 | (-2.0, -40.0, -66.0) | 0.534 | 0.039 | 0.527 | 0.189 | Left | 0.684 | 0.59 | 0.048 | 59357817271.74370 | 315.002675206244 |
| 2 | 5 | (0.0, 38.0, 30.0) | 0.064 | 0.044 | 0.207 | 0.216 | Right | 0.486 | 0.338 | 0.082 | 44585005998.11460 | 315.003132898669 |
| 2 | 6 | (-40.0, 48.0, -28.0) | 0.863 | 0.012 | 0.652 | 0.017 | Left | 0.554 | 0.577 | 0.027 | 2352347535.42941 | 315.003130481495 |
| 2 | 7 | (18.0, -100.0, -22.0) | 0.6 | 0.011 | 0.502 | 0.194 | Right | 0.752 | 0.657 | 0.023 | 5304028272.37072 | 315.003121656477 |
| 2 | 8 | (-6.0, -34.0, -62.0) | 1.386 | 0.039 | 0.54 | 0.097 | Right | 0.54 | 0.4 | 0.043 | 21048141258.54110 | 315.003079061681 |
| 2 | 9 | (-18.0, -54.0, 78.0) | 1.669 | 0.097 | 0.625 | 0.083 | Right | 0.5 | 0.515 | 0.067 | 16885669248.69490 | 315.003127331744 |
| 2 | 10 | (28.0, -100.0, -10.0) | 0.114 | 0.034 | 0.409 | 0.32 | Left | 0.468 | 0.337 | 0.07 | 38371511013.19300 | 315.003132132587 |
| 2 | 11 | (46.0, 48.0, -24.0) | 0.53 | 0.012 | 0.588 | 0.036 | Left | 0.629 | 0.536 | 0.027 | 2866508772.52007 | 315.003132781148 |
| 2 | 12 | (36.0, -52.0, 52.0) | 0.104 | 0.025 | 0.395 | 0.101 | Right | 0.505 | 0.357 | 0.091 | 43534687610.94200 | 315.003133414162 |
| 2 | 13 | (-18.0, 20.0, 70.0) | 0.903 | 0.029 | 0.373 | 0.537 | Left | 0.412 | 0.312 | 0.099 | 18290651081.57290 | 315.003132507700 |
| 2 | 14 | (-40.0, -58.0, 54.0) | 0.115 | 0.039 | 0.27 | 0.077 | Right | 0.325 | 0.17 | 0.088 | 45534397941.60060 | 315.003132740907 |
| 2 | 15 | (16.0, -36.0, 66.0) | 0.148 | 0.039 | 0.219 | 0.446 | Left | 0.43 | 0.238 | 0.109 | 42494931040.33010 | 315.003133198231 |
| 2 | 16 | (30.0, 68.0, -4.0) | 0.212 | 0.039 | 0.257 | 0.474 | Right | 0.408 | 0.292 | 0.093 | 46503826306.70200 | 315.003133041970 |
| 2 | 17 | (62.0, -8.0, 22.0) | 0.018 | 0.025 | 0.51 | 0.411 | Right | 0.56 | 0.475 | 0.049 | 32458319850.63030 | 315.003133729914 |
| 2 | 18 | (-66.0, -34.0, -4.0) | 0.059 | 0.014 | 0.607 | 0.898 | Left | 0.307 | 0.271 | 0.076 | 39163829782.24980 | 315.003131789911 |
| 2 | 19 | (-50.0, -74.0, 12.0) | 0.027 | 0.031 | 0.436 | 0.542 | Right | 0.489 | 0.381 | 0.088 | 41260426768.02160 | 315.003132113277 |
| 2 | 20 | (28.0, 42.0, 48.0) | 0.346 | 0.039 | 0.474 | 0.682 | Right | 0.433 | 0.347 | 0.073 | 6510435818.31497 | 315.003128516723 |
| 2 | 21 | (42.0, 16.0, -12.0) | 0.103 | 0.039 | 0.4 | 0.245 | Right | 0.521 | 0.461 | 0.044 | 47943819424.69710 | 315.003132404983 |
| 2 | 22 | (-6.0, -36.0, -14.0) | 0.368 | 0.014 | 0.533 | 0.274 | Left | 0.577 | 0.42 | 0.064 | 51416447703.48220 | 315.003102270956 |
| 2 | 23 | (-46.0, -64.0, 42.0) | 0.082 | 0.041 | 0.354 | 0.387 | Right | 0.315 | 0.304 | 0.073 | 42935354668.94430 | 315.003133311759 |
| 2 | 24 | (46.0, 20.0, -40.0) | 0.288 | 0.043 | 0.508 | 0.065 | Right | 0.452 | 0.413 | 0.057 | 27959904226.61030 | 315.003128535855 |
| 2 | 25 | (20.0, -28.0, -38.0) | 0.319 | 0.069 | 0.434 | 0.195 | Left | 0.382 | 0.25 | 0.044 | 39800741692.76710 | 315.003122161974 |
| 2 | 26 | (78.0, -38.0, -8.0) | 0.426 | 0.01 | 0.565 | 0.565 | Right | 0.484 | 0.387 | 0.073 | 336464993.86109 | 315.003095928108 |
| 2 | 27 | (4.0, -14.0, -24.0) | 0.056 | 0.088 | 0.437 | 0.305 | Left | 0.308 | 0.238 | 0.047 | 80439608116.98990 | 315.003124587610 |
| 2 | 28 | (50.0, -76.0, 8.0) | 0.031 | 0.031 | 0.516 | 0.084 | Right | 0.466 | 0.379 | 0.051 | 43601142799.48110 | 315.003132421222 |
| 2 | 29 | (-74.0, -14.0, 14.0) | 0.136 | 0.029 | 0.986 | 0.969 | Left | 0.132 | 0.108 | 0.085 | 477855426.00898 | 315.003131453867 |
| 2 | 30 | (10.0, -2.0, 10.0) | 0.005 | 0.039 | 0.402 | 0.342 | Right | 0.342 | 0.366 | 0.056 | 63302041435.13910 | 315.003129797841 |

Table 3. The feature set of 30 sICs for subject 3.

| Subject ID | sIC Number | sIC Center | R_o/i | Max Power Frequency | Lateralization Index | Lateralization Strength | Lateralization Side | Central Network | Clustering Coefficient | Connectivity Diversity | Central Energy | Max tIC  Non-Gaussianity |
| --- | --- | --- | --- | --- | --- | --- | --- | --- | --- | --- | --- | --- |
| 3 | 1 | (-4.0, 8.0, 72.0) | 0.489 | 0.029 | 0.432 | 0.202 | Left | 0.472 | 0.324 | 0.035 | 9990833983.58267 | 315.003126338987 |
| 3 | 2 | (0.0, -102.0, 2.0) | 0.182 | 0.012 | 0.281 | 0.449 | Right | 0.561 | 0.474 | 0.032 | 28814626803.16320 | 315.003132773711 |
| 3 | 3 | (-40.0, -54.0, 58.0) | 0.102 | 0.017 | 0.419 | 0.196 | Left | 0.426 | 0.296 | 0.046 | 40344061597.87620 | 315.003133082486 |
| 3 | 4 | (-2.0, 66.0, 20.0) | 0.058 | 0.025 | 0.558 | 0.901 | Left | 0.404 | 0.247 | 0.035 | 56802572465.39600 | 315.003129959030 |
| 3 | 5 | (-24.0, -38.0, 80.0) | 1.135 | 0.016 | 0.387 | 0.196 | Left | 0.42 | 0.31 | 0.03 | 6522809696.02804 | 315.003122021615 |
| 3 | 6 | (56.0, 16.0, 40.0) | 0.09 | 0.019 | 0.454 | 0.409 | Right | 0.398 | 0.238 | 0.042 | 35136833393.80300 | 315.003132507806 |
| 3 | 7 | (10.0, -34.0, -58.0) | 0.889 | 0.092 | 0.501 | 0.189 | Right | 0.463 | 0.52 | 0.033 | 51810085097.30350 | 315.003073774651 |
| 3 | 8 | (6.0, -84.0, 34.0) | 0.001 | 0.02 | 0.323 | 0.473 | Right | 0.537 | 0.442 | 0.033 | 38793884956.78330 | 315.003133624876 |
| 3 | 9 | (26.0, 72.0, -4.0) | 0.444 | 0.011 | 0.633 | 0.265 | Left | 0.455 | 0.385 | 0.037 | 13162504574.56050 | 315.003129614526 |
| 3 | 10 | (8.0, 62.0, 38.0) | 0.773 | 0.025 | 0.387 | 0.095 | Right | 0.466 | 0.378 | 0.027 | 12382040493.28560 | 315.003131654186 |
| 3 | 11 | (-38.0, 12.0, -22.0) | 0.14 | 0.082 | 0.389 | 0.311 | Right | 0.539 | 0.422 | 0.037 | 65050981689.61280 | 315.003115911764 |
| 3 | 12 | (-24.0, -94.0, 26.0) | 0.066 | 0.02 | 0.438 | 0.363 | Right | 0.456 | 0.258 | 0.046 | 29274455262.06570 | 315.003132262544 |
| 3 | 13 | (-64.0, -48.0, 26.0) | 0.092 | 0.017 | 0.637 | 0.588 | Left | 0.348 | 0.231 | 0.053 | 37430046193.99810 | 315.003134102643 |
| 3 | 14 | (-44.0, -80.0, -44.0) | 0.56 | 0.036 | 0.482 | 0.198 | Left | 0.399 | 0.274 | 0.034 | 8787326406.94337 | 315.003133045011 |
| 3 | 15 | (14.0, 46.0, -26.0) | 0.538 | 0.01 | 0.438 | 0.295 | Left | 0.433 | 0.309 | 0.032 | 11079225448.85740 | 315.003129848577 |
| 3 | 16 | (-46.0, -2.0, -6.0) | 0.061 | 0.082 | 0.365 | 0.142 | Left | 0.566 | 0.444 | 0.032 | 50636821796.81420 | 315.003129410070 |
| 3 | 17 | (22.0, -26.0, -40.0) | 0.366 | 0.094 | 0.406 | 0.063 | Right | 0.477 | 0.335 | 0.033 | 42053361360.65710 | 315.003119382415 |
| 3 | 18 | (62.0, 2.0, -2.0) | 0.124 | 0.01 | 0.438 | 0.049 | Right | 0.373 | 0.281 | 0.034 | 24533073772.22110 | 315.003133745862 |
| 3 | 19 | (-52.0, 4.0, 40.0) | 0.113 | 0.012 | 0.489 | 0.4 | Left | 0.37 | 0.238 | 0.044 | 31761262441.69080 | 315.003133646779 |
| 3 | 20 | (50.0, -70.0, 30.0) | 0.04 | 0.01 | 0.526 | 0.756 | Right | 0.356 | 0.268 | 0.055 | 35812705890.20340 | 315.003134360097 |
| 3 | 21 | (-14.0, -6.0, -22.0) | 0.27 | 0.094 | 0.591 | 0.171 | Right | 0.505 | 0.37 | 0.032 | 27707118020.20330 | 315.003122145810 |
| 3 | 22 | (44.0, 44.0, 26.0) | 0.051 | 0.02 | 0.643 | 0.642 | Right | 0.273 | 0.156 | 0.042 | 29413183207.78730 | 315.003129274745 |
| 3 | 23 | (-6.0, 0.0, 10.0) | 0.303 | 0.064 | 0.557 | 0.346 | Left | 0.712 | 0.608 | 0.033 | 68900575185.38480 | 315.003126000654 |
| 3 | 24 | (14.0, -38.0, 86.0) | 0.782 | 0.021 | 0.602 | 0.193 | Left | NaN | NaN | NaN | 597786090.01337 | 315.003128799614 |
| 3 | 25 | (14.0, -28.0, 88.0) | 0.671 | 0.027 | 0.846 | 1 | Right | NaN | NaN | NaN | 328578077.92158 | 315.003126216526 |
| 3 | 26 | (10.0, -110.0, 0.0) | 0.555 | 0.012 | 0.606 | 0.074 | Left | 0.627 | 0.603 | 0.029 | 2607473082.13795 | 315.003001915577 |
| 3 | 27 | (2.0, -80.0, 38.0) | 0.039 | 0.023 | 0.408 | 0.516 | Right | 0.467 | 0.285 | 0.054 | 41510748713.22420 | 315.003132542505 |
| 3 | 28 | (-4.0, -48.0, 72.0) | 0.167 | 0.01 | 0.506 | 0.185 | Left | 0.37 | 0.27 | 0.042 | 59371990268.81960 | 315.003133697669 |
| 3 | 29 | (30.0, 64.0, -20.0) | 0.451 | 0.01 | 0.656 | 0.319 | Right | 0.646 | 0.547 | 0.03 | 8566632847.72864 | 315.003070721252 |
| 3 | 30 | (-10.0, -34.0, -14.0) | 0.155 | 0.067 | 0.382 | 0.076 | Right | 0.433 | 0.269 | 0.051 | 57558954336.02750 | 315.003132137652 |

Table 4. The feature set of 30 sICs for subject 4.

| Subject ID | sIC Number | sIC Center | R_o/i | Max Power Frequency | Lateralization Index | Lateralization Strength | Lateralization Side | Central Network | Clustering Coefficient | Connectivity Diversity | Central Energy | Max tIC  Non-Gaussianity |
| --- | --- | --- | --- | --- | --- | --- | --- | --- | --- | --- | --- | --- |
| 4 | 1 | (16.0, 50.0, 46.0) | 0.473 | 0.097 | 0.38 | 0.284 | Left | 0.675 | 0.58 | 0.037 | 38166390823.82960 | 315.003130044862 |
| 4 | 2 | (-28.0, -48.0, 74.0) | 0.702 | 0.097 | 0.627 | 0.106 | Left | 0.664 | 0.608 | 0.036 | 11598906574.82840 | 315.003103350643 |
| 4 | 3 | (2.0, -80.0, 28.0) | 0.027 | 0.017 | 0.249 | 0.171 | Right | 0.56 | 0.505 | 0.036 | 61162189949.44440 | 315.003130719158 |
| 4 | 4 | (0.0, -58.0, 62.0) | 0.106 | 0.02 | 0.362 | 0.51 | Right | 0.576 | 0.418 | 0.051 | 73539495829.68930 | 315.003131230867 |
| 4 | 5 | (-42.0, 38.0, -24.0) | 0.535 | 0.097 | 0.552 | 0.047 | Right | NaN | 0.372 | 0.067 | 27164579708.75370 | 315.003128500445 |
| 4 | 6 | (0.0, -94.0, -18.0) | 0.524 | 0.016 | 0.421 | 0.132 | Right | 0.572 | 0.427 | 0.052 | 23944015096.33910 | 315.003124833318 |
| 4 | 7 | (-34.0, 60.0, 18.0) | 0.162 | 0.09 | 0.494 | 0.575 | Left | 0.504 | 0.399 | 0.06 | 49261494267.00090 | 315.003132636067 |
| 4 | 8 | (2.0, -94.0, -4.0) | 0.034 | 0.02 | 0.444 | 0.105 | Right | 0.432 | 0.385 | 0.06 | 90769882446.47570 | 315.003130429963 |
| 4 | 9 | (-62.0, -26.0, 8.0) | 0.032 | 0.027 | 0.575 | 0.69 | Left | 0.449 | 0.378 | 0.037 | 90569390750.62480 | 315.003130333806 |
| 4 | 10 | (28.0, -86.0, 22.0) | 0.065 | 0.02 | 0.473 | 0.325 | Right | 0.509 | 0.345 | 0.058 | 159275009107.15600 | 315.003133359589 |
| 4 | 11 | (36.0, -88.0, -12.0) | 0.123 | 0.033 | 0.306 | 0.361 | Right | 0.48 | 0.318 | 0.06 | 106966759353.27800 | 315.003133159231 |
| 4 | 12 | (-12.0, -4.0, 14.0) | 0.007 | 0.09 | 0.431 | 0.431 | Right | 0.569 | 0.533 | 0.048 | 20411580138.69860 | 315.003129570026 |
| 4 | 13 | (8.0, 62.0, -2.0) | 0.035 | 0.017 | 0.396 | 1 | Right | NaN | NaN | NaN | 61114511337.28450 | 315.003123890357 |
| 4 | 14 | (-34.0, -22.0, 68.0) | 0.085 | 0.015 | 0.645 | 0.98 | Left | 0.49 | 0.432 | 0.044 | 48005047173.76800 | 315.003132820672 |
| 4 | 15 | (38.0, -52.0, 64.0) | 0.061 | 0.02 | 0.718 | 1 | Right | 0.237 | 0.213 | 0.104 | 110812658071.97300 | 315.003133634571 |
| 4 | 16 | (-36.0, -68.0, 54.0) | 0.063 | 0.027 | 0.439 | 0.869 | Left | 0.302 | 0.223 | 0.076 | 176101891505.82100 | 315.003132918214 |
| 4 | 17 | (-30.0, -90.0, 22.0) | 0.054 | 0.02 | 0.615 | 0.501 | Right | 0.351 | 0.229 | 0.064 | 277403756493.61600 | 315.003133616700 |
| 4 | 18 | (68.0, -40.0, -6.0) | 0.065 | 0.027 | 0.927 | 0.938 | Right | 0.373 | 0.273 | 0.077 | 41004905733.86220 | 315.003132398301 |
| 4 | 19 | (18.0, -88.0, -32.0) | 0.612 | 0.014 | 0.814 | 0.541 | Right | 0.656 | 0.509 | 0.048 | 20556547829.18360 | 315.002604746854 |
| 4 | 20 | (-26.0, 64.0, -6.0) | 0.15 | 0.027 | 0.503 | 0.4 | Left | 0.841 | 0.863 | 0.014 | 188683282400.11000 | 5.127847932463 |
| 4 | 21 | (0.0, 58.0, 2.0) | 0.143 | 0.029 | 0.457 | 0.531 | Left | 0.382 | 0.283 | 0.055 | 94552114900.31560 | 315.003133063510 |
| 4 | 22 | (0.0, -44.0, -22.0) | 0.102 | 0.018 | 0.572 | 0.151 | Left | 0.43 | 0.361 | 0.033 | 12237370153.30950 | 315.003131184352 |
| 4 | 23 | (2.0, -38.0, -12.0) | 0.066 | 0.026 | 0.575 | 0.542 | Right | 0.487 | 0.322 | 0.05 | 14787229455.33800 | 315.003128256789 |
| 4 | 24 | (-10.0, -44.0, -58.0) | 0.444 | 0.012 | 0.61 | 0.066 | Right | 0.66 | 0.598 | 0.041 | 19475303224.20020 | 315.003036764876 |
| 4 | 25 | (-52.0, -16.0, 50.0) | 0.034 | 0.022 | 0.665 | 0.734 | Left | 0.48 | 0.402 | 0.064 | 110845888960.10100 | 315.003131448855 |
| 4 | 26 | (-2.0, -30.0, -54.0) | 0.268 | 0.069 | 0.483 | 0.11 | Right | NaN | 0.389 | 0.06 | 12696340385.22270 | 315.003067360189 |
| 4 | 27 | (-46.0, -80.0, -4.0) | 0.053 | 0.016 | 0.429 | 0.358 | Right | 0.445 | 0.276 | 0.066 | 174170568891.60200 | 315.003132650705 |
| 4 | 28 | (-4.0, -20.0, -44.0) | 0.124 | 0.043 | 0.442 | 0.131 | Right | 0.442 | 0.306 | 0.044 | 9716595606.91362 | 315.003120095162 |
| 4 | 29 | (36.0, 28.0, -36.0) | 0.138 | 0.029 | 0.708 | 0.972 | Right | 0.173 | 0.363 | 0.053 | 14584245034.95910 | 315.003133044925 |
| 4 | 30 | (64.0, -4.0, 28.0) | 0.018 | 0.02 | 0.361 | 0.638 | Right | 0.39 | 0.305 | 0.066 | 87847570724.48770 | 315.003132543803 |

Table 5. The feature set of 30 sICs for subject 5.

| Subject ID | sIC Number | sIC Center | R_o/i | Max Power Frequency | Lateralization Index | Lateralization Strength | Lateralization Side | Central Network | Clustering Coefficient | Connectivity Diversity | Central Energy | Max tIC  Non-Gaussianity |
| --- | --- | --- | --- | --- | --- | --- | --- | --- | --- | --- | --- | --- |
| 5 | 1 | (48.0, -18.0, 58.0) | 0.071 | 0.033 | 0.393 | 0.138 | Right | 0.606 | 0.468 | 0.052 | 31562220146.27450 | 315.003130760263 |
| 5 | 2 | (6.0, -84.0, 28.0) | 0.066 | 0.01 | 0.387 | 0.312 | Right | 0.668 | 0.569 | 0.037 | 35201915668.93890 | 315.003130958790 |
| 5 | 3 | (-28.0, -44.0, 74.0) | 0.456 | 0.019 | 0.72 | 0.06 | Right | 0.649 | 0.532 | 0.067 | 32812305153.60830 | 315.003120017049 |
| 5 | 4 | (-48.0, -50.0, -48.0) | 1.079 | 0.011 | 0.49 | 0.241 | Left | 0.481 | 0.411 | 0.038 | 5518970526.35596 | 315.003129164918 |
| 5 | 5 | (-12.0, -92.0, -6.0) | 0.035 | 0.01 | 0.491 | 0.444 | Left | 0.464 | 0.332 | 0.054 | 45724356561.34520 | 315.003131410185 |
| 5 | 6 | (-38.0, -78.0, 42.0) | 0.086 | 0.011 | 0.538 | 0.338 | Left | 0.416 | 0.284 | 0.07 | 33974424241.69960 | 315.003131535921 |
| 5 | 7 | (2.0, -6.0, 50.0) | 0.17 | 0.026 | 0.56 | 0.288 | Right | 0.437 | 0.265 | 0.075 | 29270184500.98370 | 315.003132548449 |
| 5 | 8 | (38.0, -58.0, 68.0) | 0.263 | 0.022 | 0.556 | 0.066 | Left | 0.43 | 0.336 | 0.058 | 25353512623.04940 | 315.003133259913 |
| 5 | 9 | (4.0, -10.0, 76.0) | 0.831 | 0.033 | 0.639 | 0.392 | Right | 0.615 | 0.46 | 0.04 | 6419959663.73817 | 315.003113272130 |
| 5 | 10 | (-10.0, -72.0, 70.0) | 1.409 | 0.018 | 0.43 | 0.377 | Left | NaN | 0.227 | 0.089 | 22041994268.28370 | 315.003129168128 |
| 5 | 11 | (28.0, -94.0, -44.0) | 1.367 | 0.011 | 0.52 | 0.319 | Right | NaN | 0.384 | 0.054 | 7167434024.78581 | 315.003131956907 |
| 5 | 12 | (0.0, -62.0, -72.0) | 1.704 | 0.033 | 0.563 | 0.045 | Right | NaN | NaN | NaN | 22063679900.56240 | 315.003120912475 |
| 5 | 13 | (40.0, -82.0, -38.0) | 0.194 | 0.01 | 0.45 | 0.167 | Left | 0.383 | 0.263 | 0.051 | 51016354409.09930 | 315.003129786024 |
| 5 | 14 | (26.0, -68.0, 58.0) | 0.193 | 0.018 | 0.69 | 0.451 | Right | 0.461 | 0.294 | 0.059 | 69211683569.62310 | 315.003132134722 |
| 5 | 15 | (-48.0, 50.0, -22.0) | 1.021 | 0.011 | 0.509 | 0.011 | Right | NaN | 0.316 | 0.03 | 11446166792.56390 | 315.003120328755 |
| 5 | 16 | (-58.0, -68.0, 20.0) | 0.224 | 0.011 | 0.714 | 0.887 | Left | 0.314 | 0.328 | 0.06 | 11698832253.44180 | 315.003131941665 |
| 5 | 17 | (-24.0, 56.0, 34.0) | 0.347 | 0.015 | 0.362 | 0.332 | Left | 0.341 | 0.211 | 0.065 | 10908901393.05040 | 315.003130968508 |
| 5 | 18 | (38.0, 48.0, 20.0) | 0.197 | 0.024 | 0.836 | 0.845 | Right | 0.371 | 0.229 | 0.053 | 37606872417.21750 | 315.003133448935 |
| 5 | 19 | (54.0, -66.0, -24.0) | 0.393 | 0.033 | 0.682 | 0.427 | Right | 0.55 | 0.455 | 0.049 | 8022695516.07024 | 315.003113798091 |
| 5 | 20 | (-50.0, -54.0, -48.0) | 0.459 | 0.011 | 0.726 | 1 | Left | 0.807 | 0.721 | 0.033 | 13715640210.75200 | 152.616597684151 |
| 5 | 21 | (54.0, -64.0, -10.0) | 0.208 | 0.022 | 0.761 | 0.732 | Right | 0.449 | 0.336 | 0.054 | 43045421039.25150 | 315.003132200546 |
| 5 | 22 | (36.0, -94.0, 0.0) | 0.115 | 0.027 | 0.457 | 0.111 | Left | 0.467 | 0.352 | 0.062 | 24470383127.66910 | 315.003132698433 |
| 5 | 23 | (10.0, -92.0, -8.0) | 0.248 | 0.033 | 0.602 | 0.323 | Right | 0.267 | 0.348 | 0.081 | 9764107205.26585 | 315.003121304580 |
| 5 | 24 | (-4.0, -22.0, 8.0) | 0.159 | 0.011 | 0.608 | 0.147 | Right | 0.447 | 0.341 | 0.036 | 16197791400.89540 | 315.003128862410 |
| 5 | 25 | (48.0, 8.0, -16.0) | 0.108 | 0.018 | 0.472 | 0.428 | Right | 0.342 | 0.309 | 0.054 | 56025389546.85490 | 315.003132664913 |
| 5 | 26 | (-48.0, -48.0, 58.0) | 0.107 | 0.024 | 0.662 | 0.826 | Left | 0.183 | 0.178 | 0.106 | 46080896859.14680 | 315.003133217386 |
| 5 | 27 | (58.0, -56.0, 32.0) | 0.156 | 0.018 | 0.705 | 0.543 | Right | 0.433 | 0.339 | 0.074 | 34834436821.58160 | 315.003132862966 |
| 5 | 28 | (-8.0, -76.0, 38.0) | 0.067 | 0.03 | 0.596 | 0.315 | Right | 0.19 | 0.216 | 0.084 | 55128065650.08580 | 315.003131668685 |
| 5 | 29 | (70.0, 20.0, 14.0) | 1.319 | 0.01 | 0.636 | 0.277 | Right | NaN | 0.408 | 0.033 | 7677835083.26615 | 315.003130775979 |
| 5 | 30 | (-2.0, -62.0, -66.0) | 1.696 | 0.093 | 0.447 | 0.475 | Right | 0.425 | 0.31 | 0.044 | 35285430180.45970 | 315.003112540013 |

Table 6. The feature set of 30 sICs for subject 6.

| Subject ID | sIC Number | sIC Center | R_o/i | Max Power Frequency | Lateralization Index | Lateralization Strength | Lateralization Side | Central Network | Clustering Coefficient | Connectivity Diversity | Central Energy | Max tIC  Non-Gaussianity |
| --- | --- | --- | --- | --- | --- | --- | --- | --- | --- | --- | --- | --- |
| 6 | 1 | (-10.0, -92.0, -14.0) | 0.52 | 0.014 | 0.412 | 0.425 | Left | NaN | 0.677 | 0.041 | 12574292869.79560 | 315.003130535121 |
| 6 | 2 | (0.0, -40.0, 50.0) | 0.112 | 0.014 | 0.53 | 0.235 | Left | 0.792 | 0.749 | 0.029 | 29373822599.36360 | 315.003131122422 |
| 6 | 3 | (-42.0, -70.0, -28.0) | 0.358 | 0.034 | 0.457 | 0.514 | Left | 0.598 | 0.502 | 0.045 | 11815314135.86690 | 315.003131330399 |
| 6 | 4 | (16.0, -82.0, 50.0) | 0.068 | 0.025 | 0.273 | 0.07 | Left | 0.442 | 0.38 | 0.075 | 38106290845.20610 | 315.003133566480 |
| 6 | 5 | (-50.0, -54.0, 54.0) | 0.272 | 0.016 | 0.579 | 0.812 | Left | 0.459 | 0.5 | 0.055 | 28519012957.51390 | 315.003133023637 |
| 6 | 6 | (-20.0, 32.0, 56.0) | 0.703 | 0.015 | 0.888 | 0.434 | Left | 0.466 | 0.491 | 0.086 | 23599524957.14510 | 304.407057694289 |
| 6 | 7 | (-4.0, -44.0, -70.0) | 2.124 | 0.052 | 0.56 | 0.229 | Right | 0.591 | 0.611 | 0.038 | 44312560373.88280 | 315.002957662235 |
| 6 | 8 | (34.0, -98.0, -2.0) | 0.146 | 0.034 | 0.35 | 0.409 | Right | 0.524 | 0.36 | 0.119 | 28527334800.69340 | 315.003132388907 |
| 6 | 9 | (38.0, -68.0, 58.0) | 0.161 | 0.021 | 0.431 | 0.712 | Right | 0.361 | 0.224 | 0.124 | 20737678354.76490 | 315.003133874345 |
| 6 | 10 | (-28.0, 4.0, 66.0) | 0.837 | 0.064 | 0.449 | 0.295 | Left | 0.308 | 0.187 | 0.156 | 25627742459.83930 | 315.003131387683 |
| 6 | 11 | (12.0, -34.0, -54.0) | 0.85 | 0.099 | 0.396 | 0.792 | Left | 0.617 | 0.496 | 0.04 | 37203554198.60370 | 315.003107757531 |
| 6 | 12 | (24.0, -38.0, 66.0) | 0.091 | 0.014 | 0.316 | 0.289 | Right | 0.3 | 0.254 | 0.1 | 49290166096.26980 | 315.003131858001 |
| 6 | 13 | (20.0, -30.0, -40.0) | 0.394 | 0.081 | 0.305 | 0.219 | Right | 0.493 | 0.336 | 0.054 | 48660220015.66190 | 315.003121418914 |
| 6 | 14 | (-4.0, -48.0, -28.0) | 0.072 | 0.014 | 0.482 | 0.197 | Left | 0.46 | 0.402 | 0.031 | 36808795934.50550 | 315.003130196607 |
| 6 | 15 | (58.0, -60.0, -22.0) | 0.336 | 0.029 | 0.858 | 0.867 | Right | 0.428 | 0.313 | 0.065 | 20997296092.42130 | 315.003132969272 |
| 6 | 16 | (-20.0, 60.0, -18.0) | 0.684 | 0.025 | 0.692 | 0.356 | Left | 0.481 | 0.307 | 0.038 | 34615853454.06730 | 315.003129249892 |
| 6 | 17 | (-54.0, 26.0, -6.0) | 0.189 | 0.027 | 0.829 | 0.944 | Left | 0.363 | 0.266 | 0.049 | 42803728111.47780 | 315.003133696123 |
| 6 | 18 | (-14.0, 60.0, 0.0) | 0.342 | 0.02 | 0.806 | 0.212 | Right | 0.61 | 0.47 | 0.049 | 55438869605.64620 | 315.003056180486 |
| 6 | 19 | (-54.0, -60.0, -24.0) | 0.984 | 0.016 | 0.705 | 0.771 | Left | 0.322 | 0.38 | 0.057 | 3185758790.34233 | 315.003129914811 |
| 6 | 20 | (26.0, 46.0, -50.0) | 0.718 | 0.025 | 0.658 | 0.237 | Right | 0.626 | 0.422 | 0.076 | 86247103069.99100 | 312.809177358090 |
| 6 | 21 | (26.0, 40.0, -40.0) | 1.558 | 0.01 | 0.707 | 0.307 | Right | 0.698 | 0.528 | 0.11 | 15210809725.88550 | 34.985867317644 |
| 6 | 22 | (-28.0, -14.0, -44.0) | 0.453 | 0.014 | 0.59 | 0.304 | Right | 0.495 | 0.337 | 0.03 | 6090974375.02721 | 315.003131102773 |
| 6 | 23 | (-54.0, -8.0, 36.0) | 0.054 | 0.039 | 0.402 | 0.02 | Left | 0.529 | 0.367 | 0.109 | 70932290239.87990 | 315.003132307972 |
| 6 | 24 | (62.0, -48.0, 6.0) | 0.085 | 0.025 | 0.459 | 0.163 | Right | 0.283 | 0.246 | 0.082 | 38098725122.30450 | 315.003133941884 |
| 6 | 25 | (6.0, -42.0, -12.0) | 0.089 | 0.097 | 0.464 | 0.048 | Right | 0.499 | 0.351 | 0.04 | 52304771403.30260 | 315.003129650071 |
| 6 | 26 | (-28.0, 56.0, 28.0) | 0.127 | 0.021 | 0.624 | 0.218 | Right | 0.438 | 0.35 | 0.05 | 73364771169.86520 | 315.003131953666 |
| 6 | 27 | (58.0, 10.0, 10.0) | 0.137 | 0.016 | 0.54 | 0.893 | Right | 0.396 | 0.312 | 0.055 | 107170039687.60600 | 315.003133132956 |
| 6 | 28 | (20.0, 38.0, -42.0) | 0.286 | 0.014 | 0.84 | 0.28 | Right | 0.593 | 0.459 | 0.127 | 4762856859.65926 | 112.711876677865 |
| 6 | 29 | (-62.0, -30.0, -14.0) | 0.024 | 0.016 | 0.638 | 1 | Left | 0.246 | 0.244 | 0.068 | 35346065224.17050 | 315.003133540888 |
| 6 | 30 | (28.0, 34.0, -44.0) | 1.004 | 0.011 | 0.817 | 0.951 | Right | 0.441 | 0.315 | 0.091 | 8351728851.80092 | 315.002088316823 |

Table 7. The feature set of 30 sICs for subject 7.

| Subject ID | sIC Number | sIC Center | R_o/i | Max Power Frequency | Lateralization Index | Lateralization Strength | Lateralization Side | Central Network | Clustering Coefficient | Connectivity Diversity | Central Energy | Max tIC  Non-Gaussianity |
| --- | --- | --- | --- | --- | --- | --- | --- | --- | --- | --- | --- | --- |
| 7 | 1 | (-32.0, 66.0, 2.0) | 1.309 | 0.014 | 0.395 | 0.115 | Right | NaN | 0.502 | 0.044 | 47136354498.69000 | 315.003117357357 |
| 7 | 2 | (-32.0, -82.0, -30.0) | 0.754 | 0.016 | 0.57 | 0.318 | Left | 0.738 | 0.68 | 0.018 | 23559470822.43120 | 315.003076898611 |
| 7 | 3 | (30.0, 16.0, 66.0) | 0.225 | 0.019 | 0.354 | 0.846 | Right | 0.511 | 0.453 | 0.044 | 207811835551.59300 | 315.003125473531 |
| 7 | 4 | (44.0, 50.0, 12.0) | 0.108 | 0.016 | 0.488 | 0.854 | Right | 0.544 | 0.386 | 0.101 | 112253906910.93700 | 315.003113161157 |
| 7 | 5 | (-8.0, -84.0, 44.0) | 0.021 | 0.016 | 0.284 | 0.285 | Left | 0.392 | 0.446 | 0.061 | 115114110722.98300 | 315.003128389504 |
| 7 | 6 | (34.0, -16.0, -42.0) | 0.459 | 0.019 | 0.512 | 0.742 | Left | 0.804 | 0.779 | 0.019 | 7346566796.11640 | 315.003114762192 |
| 7 | 7 | (22.0, -104.0, -6.0) | 0.24 | 0.019 | 0.314 | 0.264 | Left | 0.586 | 0.416 | 0.059 | 120675924269.48100 | 315.003131741123 |
| 7 | 8 | (48.0, -48.0, 56.0) | 0.072 | 0.016 | 0.468 | 0.603 | Right | 0.34 | 0.326 | 0.093 | 222209844680.16500 | 315.003131618083 |
| 7 | 9 | (-2.0, 62.0, 24.0) | 0.265 | 0.016 | 0.492 | 0.077 | Right | 0.382 | 0.438 | 0.108 | 147379758733.74700 | 315.003126746996 |
| 7 | 10 | (12.0, 10.0, 76.0) | 2.073 | 0.033 | 0.58 | 0.046 | Left | NaN | NaN | NaN | 38907869802.58520 | 315.003054195606 |
| 7 | 11 | (-8.0, 58.0, 36.0) | 0.193 | 0.054 | 0.273 | 0.575 | Right | 0.514 | 0.367 | 0.053 | 192831035420.19900 | 315.003131814576 |
| 7 | 12 | (12.0, -80.0, 52.0) | 0.034 | 0.016 | 0.387 | 0.273 | Right | 0.504 | 0.417 | 0.045 | 148120789113.32400 | 315.003131277623 |
| 7 | 13 | (-34.0, 10.0, 68.0) | 1.588 | 0.033 | 0.463 | 0.165 | Right | NaN | 0.337 | 0.065 | 9324957413.52821 | 315.003115373828 |
| 7 | 14 | (30.0, 64.0, -4.0) | 0.116 | 0.014 | 0.298 | 0.585 | Right | 0.481 | 0.383 | 0.075 | 314726910853.40700 | 315.003130208361 |
| 7 | 15 | (-48.0, -60.0, -56.0) | 1.833 | 0.016 | 0.561 | 0.117 | Left | NaN | 0.549 | 0.044 | 6021269473.21807 | 315.003129315159 |
| 7 | 16 | (24.0, -74.0, -32.0) | 0.259 | 0.016 | 0.268 | 0.588 | Right | 0.466 | 0.469 | 0.049 | 27025808600.83760 | 315.003130306618 |
| 7 | 17 | (0.0, -48.0, 74.0) | 0.345 | 0.016 | 0.431 | 0.426 | Right | 0.251 | 0.343 | 0.113 | 79839909525.86540 | 315.003122599993 |
| 7 | 18 | (-22.0, -108.0, -10.0) | 0.364 | 0.019 | 0.476 | 0.643 | Right | NaN | NaN | NaN | 32550011376.73470 | 315.003106708051 |
| 7 | 19 | (4.0, -72.0, 60.0) | 0.671 | 0.016 | 0.584 | 0.387 | Right | 0.786 | 0.781 | 0.014 | 23089254219.97900 | 315.002616543375 |
| 7 | 20 | (40.0, 56.0, -22.0) | 0.782 | 0.016 | 0.507 | 0.328 | Right | 0.515 | 0.472 | 0.061 | 18726724698.52400 | 315.003122507650 |
| 7 | 21 | (64.0, -18.0, 42.0) | 0.074 | 0.019 | 0.518 | 0.492 | Right | 0.489 | 0.426 | 0.064 | 231815794601.92400 | 315.003128736606 |
| 7 | 22 | (-64.0, 20.0, 22.0) | 0.41 | 0.016 | 0.85 | 0.836 | Left | 0.801 | 0.703 | 0.022 | 13638289197.91480 | 270.794950018061 |
| 7 | 23 | (20.0, -26.0, 28.0) | 0.004 | 0.016 | 0.38 | 0.612 | Right | 0.643 | 0.579 | 0.038 | 35622937660.45420 | 315.003130202371 |
| 7 | 24 | (54.0, -60.0, -44.0) | 1.988 | 0.016 | 0.874 | 0.525 | Right | 0.561 | 0.476 | 0.047 | 31080539474.31480 | 315.003127953151 |
| 7 | 25 | (68.0, -50.0, -6.0) | 0.101 | 0.016 | 0.561 | 0.068 | Left | 0.379 | 0.322 | 0.062 | 51050153171.49700 | 315.003128535751 |
| 7 | 26 | (-50.0, -44.0, 56.0) | 0.184 | 0.016 | 0.9 | 0.976 | Left | 0.336 | 0.238 | 0.08 | 409992669118.26000 | 315.003130300942 |
| 7 | 27 | (-60.0, 16.0, 34.0) | 0.11 | 0.016 | 0.654 | 0.985 | Left | 0.437 | 0.399 | 0.042 | 90546776498.65900 | 315.003131479812 |
| 7 | 28 | (50.0, -72.0, 30.0) | 0.024 | 0.018 | 0.375 | 0.227 | Right | 0.476 | 0.497 | 0.047 | 107295794206.20300 | 315.003132490012 |
| 7 | 29 | (10.0, -16.0, 12.0) | 0.029 | 0.016 | 0.399 | 0.141 | Right | 0.612 | 0.439 | 0.067 | 20974883110.22240 | 315.003131064934 |
| 7 | 30 | (-40.0, -20.0, -8.0) | 0.076 | 0.016 | 0.383 | 0.032 | Left | 0.433 | 0.374 | 0.046 | 22086093903.99050 | 315.003129885005 |

Table 8. The feature set of 30 sICs for subject 8.

| Subject ID | sIC Number | sIC Center | R_o/i | Max Power Frequency | Lateralization Index | Lateralization Strength | Lateralization Side | Central Network | Clustering Coefficient | Connectivity Diversity | Central Energy | Max tIC  Non-Gaussianity |
| --- | --- | --- | --- | --- | --- | --- | --- | --- | --- | --- | --- | --- |
| 8 | 1 | (8.0, -94.0, -30.0) | 0.937 | 0.036 | 0.413 | 0.028 | Right | NaN | 0.597 | 0.03 | 6912324929.81782 | 315.003128687846 |
| 8 | 2 | (0.0, -44.0, 62.0) | 0.14 | 0.017 | 0.413 | 0.156 | Right | 0.37 | 0.276 | 0.054 | 40023148769.67380 | 315.003129063333 |
| 8 | 3 | (-14.0, -94.0, 32.0) | 0.24 | 0.019 | 0.422 | 0.107 | Left | 0.522 | 0.34 | 0.044 | 22828446132.56430 | 315.003131724916 |
| 8 | 4 | (58.0, 16.0, -2.0) | 0.217 | 0.034 | 0.521 | 0.578 | Right | 0.402 | 0.261 | 0.059 | 83131683704.61460 | 315.003130164952 |
| 8 | 5 | (-50.0, -72.0, 32.0) | 0.138 | 0.021 | 0.445 | 0.161 | Left | 0.594 | 0.425 | 0.053 | 17802856398.70490 | 315.003132197848 |
| 8 | 6 | (-18.0, -28.0, -38.0) | 0.214 | 0.099 | 0.468 | 0.603 | Left | 0.453 | 0.307 | 0.044 | 27162954719.01820 | 315.003123276304 |
| 8 | 7 | (-46.0, -4.0, -50.0) | 0.942 | 0.023 | 0.59 | 0.201 | Left | 0.401 | 0.323 | 0.029 | 25196816269.10540 | 315.003130559749 |
| 8 | 8 | (0.0, -92.0, 4.0) | 0.105 | 0.02 | 0.349 | 0.595 | Left | 0.49 | 0.29 | 0.046 | 25876031343.79410 | 315.003130480873 |
| 8 | 9 | (-60.0, 6.0, 20.0) | 0.037 | 0.077 | 0.589 | 0.385 | Left | 0.469 | 0.23 | 0.039 | 174871776664.33800 | 315.003131582203 |
| 8 | 10 | (-6.0, -70.0, 54.0) | 0.214 | 0.019 | 0.498 | 0.502 | Left | 0.433 | 0.237 | 0.063 | 46717388044.86480 | 315.003131298091 |
| 8 | 11 | (-24.0, 32.0, 54.0) | 0.265 | 0.015 | 0.623 | 0.829 | Left | 0.359 | 0.257 | 0.048 | 43174809694.73470 | 315.003131972884 |
| 8 | 12 | (36.0, 56.0, 20.0) | 0.183 | 0.019 | 0.566 | 0.188 | Left | 0.495 | 0.389 | 0.038 | 50357763292.41290 | 315.003129198740 |
| 8 | 13 | (-54.0, -58.0, -22.0) | 0.343 | 0.019 | 0.827 | 0.261 | Left | 0.471 | 0.405 | 0.039 | 2858310047.91616 | 315.003126363169 |
| 8 | 14 | (-24.0, -26.0, -48.0) | 1.102 | 0.042 | 0.673 | 0.102 | Left | 0.502 | 0.386 | 0.04 | 5833215300.35019 | 315.003103349106 |
| 8 | 15 | (-30.0, -58.0, 68.0) | 0.657 | 0.023 | 0.725 | 0.86 | Left | 0.419 | 0.342 | 0.059 | 65581961352.96410 | 315.003131545207 |
| 8 | 16 | (-12.0, -12.0, -54.0) | 1.509 | 0.078 | 0.687 | 0.125 | Left | NaN | 0.377 | 0.039 | 2850008547.76116 | 315.003019978001 |
| 8 | 17 | (-6.0, -48.0, -72.0) | 1.167 | 0.014 | 0.724 | 0.346 | Left | NaN | NaN | NaN | 74797592507.06720 | 315.003120029690 |
| 8 | 18 | (-18.0, -86.0, 56.0) | 1.01 | 0.016 | 0.581 | 0.032 | Right | 0.524 | 0.543 | 0.042 | 1866847390.99206 | 315.000516042676 |
| 8 | 19 | (-38.0, -92.0, -12.0) | 0.309 | 0.012 | 0.409 | 0.418 | Left | NaN | 0.28 | 0.05 | 7194961423.17141 | 315.003131144033 |
| 8 | 20 | (-6.0, 4.0, 62.0) | 0.088 | 0.015 | 0.585 | 0.632 | Left | 0.367 | 0.271 | 0.052 | 80423619398.98990 | 315.003131009501 |
| 8 | 21 | (8.0, -64.0, -72.0) | 1.153 | 0.098 | 0.627 | 0.178 | Right | NaN | NaN | NaN | 38459097443.61930 | 315.003125629887 |
| 8 | 22 | (44.0, 38.0, 34.0) | 0.052 | 0.023 | 0.618 | 0.812 | Right | 0.384 | 0.255 | 0.039 | 48980904648.84260 | 315.003132454243 |
| 8 | 23 | (12.0, -22.0, -50.0) | 0.86 | 0.042 | 0.814 | 0.106 | Right | 0.25 | 0.373 | 0.04 | 15578820827.01630 | 315.003040969128 |
| 8 | 24 | (-16.0, -2.0, 70.0) | 0.918 | 0.02 | 0.629 | 0.721 | Left | 0.272 | 0.185 | 0.05 | 29635489310.94750 | 315.003129884884 |
| 8 | 25 | (52.0, -54.0, 54.0) | 0.201 | 0.012 | 0.757 | 0.855 | Right | 0.291 | 0.188 | 0.069 | 42948102946.90660 | 315.003133189517 |
| 8 | 26 | (-6.0, -6.0, -26.0) | 0.456 | 0.084 | 0.693 | 0.266 | Left | 0.768 | 0.645 | 0.038 | 30519482409.95520 | 46.453011100244 |
| 8 | 27 | (-6.0, -70.0, 48.0) | 0.092 | 0.019 | 0.552 | 0.844 | Left | 0.471 | 0.294 | 0.074 | 47237135049.81180 | 315.003132475845 |
| 8 | 28 | (30.0, 48.0, -38.0) | 4.197 | 0.014 | 0.776 | 0.106 | Right | NaN | 0.543 | 0.034 | 10515283946.83480 | 315.002605558469 |
| 8 | 29 | (-52.0, -56.0, 48.0) | 0.207 | 0.019 | 0.776 | 0.917 | Left | 0.376 | 0.238 | 0.069 | 35201606770.10150 | 315.003133880092 |
| 8 | 30 | (-6.0, -32.0, 2.0) | 0.219 | 0.098 | 0.58 | 0.615 | Left | 0.384 | 0.233 | 0.039 | 31029198634.32340 | 315.003130228683 |

Table 9. The feature set of 30 sICs for subject 9.

| Subject ID | sIC Number | sIC Center | R_o/i | Max Power Frequency | Lateralization Index | Lateralization Strength | Lateralization Side | Central Network | Clustering Coefficient | Connectivity Diversity | Central Energy | Max tIC  Non-Gaussianity |
| --- | --- | --- | --- | --- | --- | --- | --- | --- | --- | --- | --- | --- |
| 9 | 1 | (18.0, -88.0, -12.0) | 0.522 | 0.015 | 0.517 | 0.709 | Right | NaN | 0.36 | 0.046 | 12112410907.44260 | 315.003131554880 |
| 9 | 2 | (-46.0, 30.0, 26.0) | 0.141 | 0.011 | 0.61 | 0.717 | Left | 0.554 | 0.381 | 0.04 | 50643783095.67480 | 315.003131196872 |
| 9 | 3 | (-12.0, 42.0, 52.0) | 0.29 | 0.027 | 0.632 | 0.355 | Left | 0.503 | 0.418 | 0.045 | 23743208307.01170 | 315.003130555001 |
| 9 | 4 | (56.0, -62.0, 28.0) | 0.146 | 0.025 | 0.409 | 0.203 | Right | 0.519 | 0.392 | 0.053 | 36961960155.83290 | 315.003133982500 |
| 9 | 5 | (46.0, 30.0, 38.0) | 0.216 | 0.01 | 0.981 | 0.639 | Right | 0.529 | 0.424 | 0.047 | 21516734314.46010 | 315.003132673965 |
| 9 | 6 | (-58.0, -30.0, 52.0) | 0.348 | 0.011 | 0.478 | 0.136 | Right | 0.455 | 0.342 | 0.048 | 47917589847.78210 | 315.003132612988 |
| 9 | 7 | (2.0, -78.0, 16.0) | 0.067 | 0.042 | 0.372 | 0.515 | Right | 0.501 | 0.408 | 0.036 | 31607106713.45500 | 315.003132610946 |
| 9 | 8 | (-2.0, -100.0, 0.0) | 0.058 | 0.019 | 0.456 | 0.161 | Left | 0.569 | 0.465 | 0.039 | 29353707915.88740 | 315.003132327288 |
| 9 | 9 | (64.0, 6.0, 4.0) | 0.198 | 0.026 | 0.494 | 0.317 | Right | 0.437 | 0.338 | 0.04 | 17958658965.28460 | 315.003133198996 |
| 9 | 10 | (-4.0, -58.0, 64.0) | 0.138 | 0.015 | 0.48 | 0.043 | Right | 0.392 | 0.27 | 0.059 | 45278492798.32750 | 315.003132918403 |
| 9 | 11 | (62.0, 30.0, 2.0) | 0.655 | 0.012 | 0.921 | 0.924 | Right | 0.449 | 0.333 | 0.042 | 6807138350.19327 | 315.003132752046 |
| 9 | 12 | (58.0, -42.0, 52.0) | 0.172 | 0.011 | 0.551 | 0.262 | Right | 0.376 | 0.275 | 0.103 | 39039585736.84860 | 315.003130876161 |
| 9 | 13 | (-14.0, -72.0, 60.0) | 0.146 | 0.015 | 0.514 | 0.285 | Right | 0.35 | 0.272 | 0.071 | 47638907919.87220 | 315.003132526476 |
| 9 | 14 | (18.0, 68.0, -20.0) | 0.739 | 0.019 | 0.657 | 0.746 | Right | 0.423 | 0.297 | 0.044 | 5665951839.48642 | 315.003129801328 |
| 9 | 15 | (-22.0, -44.0, 82.0) | 0.295 | 0.024 | 0.742 | 0.604 | Left | 0.08 | 0.131 | 0.089 | 12609534.58141 | 315.003126433931 |
| 9 | 16 | (-62.0, 22.0, 12.0) | 0.918 | 0.011 | 0.937 | 0.195 | Left | NaN | NaN | NaN | 5514371331.26099 | 315.003128064869 |
| 9 | 17 | (4.0, -12.0, 76.0) | 0.727 | 0.015 | 0.953 | 0.219 | Right | 0.476 | 0.557 | 0.035 | 16327524053.50040 | 315.003104998668 |
| 9 | 18 | (14.0, -96.0, -22.0) | 1.05 | 0.015 | 0.718 | 0.766 | Right | NaN | 0.371 | 0.063 | 8541745168.88492 | 315.003132278368 |
| 9 | 19 | (30.0, -66.0, -66.0) | 0.952 | 0.01 | 0.716 | 0.001 | Right | 0.458 | 0.332 | 0.04 | 9473828498.13596 | 315.003131309914 |
| 9 | 20 | (-54.0, 4.0, 42.0) | 0.122 | 0.011 | 0.709 | 0.62 | Left | 0.422 | 0.39 | 0.041 | 80926371186.47950 | 315.003131671766 |
| 9 | 21 | (40.0, 58.0, 10.0) | 0.156 | 0.01 | 0.573 | 0.465 | Right | 0.517 | 0.391 | 0.051 | 29053809847.29700 | 315.003126885530 |
| 9 | 22 | (54.0, 16.0, -8.0) | 0.32 | 0.011 | 0.6 | 0.326 | Right | 0.393 | 0.286 | 0.061 | 36537894718.52190 | 315.003132883214 |
| 9 | 23 | (4.0, -92.0, -24.0) | 0.276 | 0.011 | 0.603 | 0.016 | Right | 0.311 | 0.155 | 0.065 | 13432455941.40120 | 315.003132583289 |
| 9 | 24 | (60.0, 16.0, 28.0) | 0.123 | 0.01 | 0.626 | 0.664 | Right | 0.24 | 0.195 | 0.055 | 47161185059.77180 | 315.003133155485 |
| 9 | 25 | (-14.0, -34.0, -60.0) | 0.783 | 0.089 | 0.631 | 0.196 | Right | 0.526 | 0.351 | 0.03 | 5259104970.86671 | 315.003108963700 |
| 9 | 26 | (-8.0, 44.0, -30.0) | 0.909 | 0.011 | 0.581 | 0.001 | Left | 0.359 | 0.339 | 0.041 | 9091193181.79206 | 315.003132141205 |
| 9 | 27 | (18.0, -18.0, -44.0) | 0.499 | 0.097 | 0.67 | 0.542 | Right | NaN | 0.288 | 0.039 | 3710945638.76500 | 315.003123045206 |
| 9 | 28 | (52.0, -56.0, -24.0) | 0.332 | 0.017 | 0.682 | 0.725 | Right | 0.653 | 0.573 | 0.041 | 13649664421.46940 | 315.003125509829 |
| 9 | 29 | (2.0, -70.0, -68.0) | 0.453 | 0.09 | 0.731 | 0.015 | Right | 0.526 | 0.377 | 0.055 | 8409908360.98689 | 315.003096263661 |
| 9 | 30 | (-4.0, -40.0, -34.0) | 0.08 | 0.011 | 0.566 | 0.381 | Left | 0.433 | 0.346 | 0.034 | 23360497994.93190 | 315.003131987882 |

Table 10. The feature set of 30 sICs for subject 10.

| Subject ID | sIC Number | sIC Center | R_o/i | Max Power Frequency | Lateralization Index | Lateralization Strength | Lateralization Side | Central Network | Clustering Coefficient | Connectivity Diversity | Central Energy | Max tIC  Non-Gaussianity |
| --- | --- | --- | --- | --- | --- | --- | --- | --- | --- | --- | --- | --- |
| 10 | 1 | (6.0, -62.0, -4.0) | 0.046 | 0.092 | 0.278 | 0.32 | Left | 0.661 | 0.498 | 0.048 | 60090091972.99310 | 315.003131344512 |
| 10 | 2 | (-12.0, -22.0, 84.0) | 0.743 | 0.084 | 0.358 | 0.013 | Left | 0.636 | 0.563 | 0.036 | 57021219461.35660 | 315.003129679491 |
| 10 | 3 | (-30.0, -60.0, -58.0) | 0.338 | 0.011 | 0.337 | 0.153 | Left | 0.513 | 0.369 | 0.076 | 42365245377.19080 | 315.003129135145 |
| 10 | 4 | (68.0, -22.0, -8.0) | 0.015 | 0.034 | 0.96 | 1 | Right | 0.46 | 0.317 | 0.062 | 39104325304.15020 | 315.003132864746 |
| 10 | 5 | (14.0, -52.0, 6.0) | 0.071 | 0.024 | 0.399 | 0.601 | Right | 0.439 | 0.441 | 0.056 | 27260258891.88650 | 315.003130871351 |
| 10 | 6 | (-2.0, -12.0, 80.0) | 0.376 | 0.011 | 0.544 | 0.49 | Right | 0.643 | 0.64 | 0.022 | 24263847221.25910 | 315.003126349916 |
| 10 | 7 | (8.0, -80.0, 46.0) | 0.127 | 0.026 | 0.395 | 0.114 | Right | 0.567 | 0.504 | 0.038 | 54536892861.78150 | 315.003132243833 |
| 10 | 8 | (-24.0, -94.0, 16.0) | 0.156 | 0.079 | 0.29 | 0.097 | Left | 0.53 | 0.451 | 0.044 | 26353843766.28480 | 315.003131930129 |
| 10 | 9 | (68.0, -28.0, 36.0) | 0.101 | 0.01 | 0.58 | 0.976 | Right | 0.466 | 0.346 | 0.071 | 38163829286.53290 | 315.003133699605 |
| 10 | 10 | (64.0, 20.0, 8.0) | 0.115 | 0.027 | 0.407 | 0.805 | Right | 0.41 | 0.417 | 0.066 | 8724295223.41954 | 315.003133076034 |
| 10 | 11 | (18.0, 74.0, 16.0) | 0.461 | 0.028 | 0.479 | 0.228 | Right | 0.556 | 0.386 | 0.058 | 7762943542.97873 | 315.003131631560 |
| 10 | 12 | (10.0, 46.0, 44.0) | 0.051 | 0.046 | 0.322 | 0.109 | Right | 0.402 | 0.374 | 0.05 | 42846004646.45910 | 315.003126167231 |
| 10 | 13 | (-26.0, -56.0, -68.0) | 1.633 | 0.043 | 0.517 | 0.038 | Right | 0.467 | 0.334 | 0.057 | 3219838877.79142 | 315.003126911975 |
| 10 | 14 | (48.0, 60.0, -22.0) | 0.787 | 0.044 | 0.444 | 0.755 | Right | 0.389 | 0.336 | 0.097 | 2754340039.81930 | 315.003127950159 |
| 10 | 15 | (-64.0, -58.0, 22.0) | 0.168 | 0.043 | 0.752 | 0.905 | Left | 0.633 | 0.545 | 0.027 | 18700432308.43150 | 315.003132972216 |
| 10 | 16 | (62.0, -58.0, 16.0) | 0.766 | 0.031 | 0.575 | 0.582 | Right | 0.486 | 0.518 | 0.046 | 21903620284.28260 | 315.003132762855 |
| 10 | 17 | (-12.0, 0.0, -24.0) | 0.158 | 0.079 | 0.528 | 0.483 | Left | 0.527 | 0.452 | 0.042 | 67836541759.24330 | 315.003127886197 |
| 10 | 18 | (-24.0, -66.0, 64.0) | 0.275 | 0.01 | 0.463 | 0.144 | Right | 0.545 | 0.549 | 0.035 | 11221616271.91120 | 315.003132549447 |
| 10 | 19 | (-38.0, -84.0, -16.0) | 0.753 | 0.042 | 0.623 | 0.128 | Right | 0.618 | 0.548 | 0.037 | 14791224700.27780 | 315.003105501237 |
| 10 | 20 | (-20.0, -94.0, -40.0) | 0.833 | 0.01 | 0.541 | 0.742 | Left | 0.542 | 0.429 | 0.045 | 3316529460.92811 | 315.003127218623 |
| 10 | 21 | (58.0, -16.0, 56.0) | 1.016 | 0.012 | 0.42 | 0.508 | Right | 0.54 | 0.45 | 0.049 | 5974898091.99828 | 315.003128676842 |
| 10 | 22 | (34.0, 40.0, -28.0) | 1.085 | 0.095 | 0.658 | 0.298 | Right | 0.471 | 0.377 | 0.067 | 1590533792.69511 | 315.003128395648 |
| 10 | 23 | (60.0, -18.0, -36.0) | 0.434 | 0.012 | 0.602 | 0.179 | Right | 0.098 | 0.277 | 0.126 | 8955911456.21781 | 315.003130181572 |
| 10 | 24 | (34.0, -60.0, 68.0) | 2.014 | 0.042 | 0.516 | 0.28 | Right | 0.63 | 0.595 | 0.031 | 2296623100.80550 | 315.003128543730 |
| 10 | 25 | (-10.0, -92.0, -34.0) | 0.572 | 0.01 | 0.49 | 0.05 | Right | 0.636 | 0.52 | 0.062 | 31469723298.24400 | 315.003130167572 |
| 10 | 26 | (-64.0, -2.0, -6.0) | 0.032 | 0.019 | 0.536 | 0.792 | Left | 0.261 | 0.211 | 0.13 | 11346517702.95870 | 315.003131140200 |
| 10 | 27 | (-10.0, -32.0, -46.0) | 0.407 | 0.044 | 0.505 | 0.591 | Right | 0.516 | 0.376 | 0.043 | 43630938681.50660 | 315.003126795626 |
| 10 | 28 | (-12.0, -42.0, -70.0) | 3.86 | 0.085 | 0.534 | 0.567 | Right | 0.422 | 0.342 | 0.07 | 26999996354.69020 | 315.003087811100 |
| 10 | 29 | (-44.0, 28.0, 44.0) | 0.461 | 0.044 | 0.654 | 0.13 | Left | 0.785 | 0.739 | 0.024 | 4077367074.45139 | 213.210486177606 |
| 10 | 30 | (14.0, -36.0, -12.0) | 0.283 | 0.065 | 0.496 | 0.364 | Right | 0.65 | 0.577 | 0.035 | 51311587928.99490 | 315.003127488126 |
